# New skeletal material sheds light on the palaeobiology of the Pleistocene marsupial carnivore, *Thylacoleo carnifex*

**DOI:** 10.1371/journal.pone.0208020

**Published:** 2018-12-12

**Authors:** Roderick T. Wells, Aaron B. Camens

**Affiliations:** 1 Ecology and Evolution, College of Science and Engineering, Flinders University, Adelaide, South Australia, Australia; 2 Palaeontology, South Australian Museum, Adelaide, South Australia, Australia; Monash University, AUSTRALIA

## Abstract

The extinct marsupial ‘lion’ *Thylacoleo carnifex* was Australia’s largest mammalian carnivore. Despite being the topic of more discussion than any other extinct Australian marsupial (excepting perhaps the Thylacine), basic aspects of its palaeobiology, including its locomotory repertoire, remain poorly understood. Recent discoveries allowed the first reconstruction of an entire skeleton including the first complete tail and hitherto-unrecognised clavicles. Here we describe these elements and re-assess the biomechanics of the postcranial skeleton via comparisons with a range of extant terrestrial, scansorial and arboreal Australian marsupials. Our analysis suggests that *T*. *carnifex* possessed: a relatively stiff tail comprising half of the vertebral column length; proximal caudal centra exhibiting a relatively high resistance to sagittal and lateral bending (RSB and RTB); relatively enlarged areas of origin for caudal flexors and extensors; a rigid lumbar spine; and a shoulder girdle braced by strong clavicles. The lever arms of major muscle/tendon systems controlling the axial and appendicular skeleton were identified and RSB and RTB calculated. The combination of these features compared most closely overall with those of the much smaller Tasmanian Devil (*Sarcophilus harrisii*), a hunter/scavenger capable of climbing. Similar locomotor behaviour is proposed for *Thylacoleo carnifex*. Orientation of articular facets and RSB stresses also indicate that *T*. *carnifex* may have held its tail in a dorsally-flexed position.

## Introduction

“One of the fellest and most destructive of predatory beasts” was the descriptor used by Richard Owen when discussing the fossilised remains of the marsupial ‘lion’, *Thylacoleo carnifex* [1]. Owen’s interpretation was based on the carnassial premolars, particularly the P^3^. The diprotodont incisor dentition, the vestigial nature of the canines and the absence of any significant associated postcranial material led others to challenge Owen’s carnivore hypothesis [2, 3]. Owen’s interpretation gained further support with discovery of the first articulated skeletal remains from a loam pit near Moree in NSW in 1966. This heavily gypsum-encrusted specimen allowed some general comparisons of skeletal proportions to be made with those of extant marsupial and eutherian carnivores and omnivores [4, 5, 6]. Further skeletal remains were recovered from cave sites at Naracoorte in South Australia in the 1970s leading to the description of the manus and a partial pes [7] and a reconstruction of the jaw mechanics [8]. Either missing or perhaps unrecognised from all these finds were the caudal vertebrae, distal portions of the pes and the clavicles.

Blasting at Henschke’s Quarry, Naracoorte, in 2007 exposed a cavern containing near-complete remains of a number of individuals including partially-articulated vertebral columns, limbs and hind feet [9]. Around this time an isolated, complete *T*. *carnifex* skeleton was discovered in a cave beneath the Nullarbor Plain [10]. Subsequently additional skeletal remains were recovered from other caves in the same region. These well preserved Nullarbor and Naracoorte specimens provided an opportunity to reassemble and review the structural and functional aspects of the entire skeleton.

In this paper we describe the hitherto missing elements and revisit the related features of the lumbar vertebrae, sacrum and pelvis first described by Finch [4] and Finch and Freedman [5]. We describe the clavicles and reappraise the structure of the axial skeleton, including the pelvic and pectoral girdles. Following on from Finch [4], we combine measurements of all vertebral processes along with published work and whole body x-rays of extant marsupial species by Vogelnest & Allan [11] to propose an anatomical and functional reconstruction of posture and locomotion in *T*. *carnifex*. Functional inferences are based on comparison with limb and vertebral proportions of extant Australian marsupials in which behaviour is known.

Inferring behaviour from skeletal structure is underpinned by the notion that form reflects function [12,13]. However, form represents a compromise between the constraints of ancestry, morphogenesis and environment; to this end thylacoleonids pose an interesting challenge. A unique lineage, clearly derived from a diprotodontian ancestry, they appear to have evolved towards hyper-carnivory [8, 14, 15, 16]. Perhaps not surprisingly the skeleton of *T*. *carnifex* presents more as an amalgam of different features found in a range of extant marsupials. Of particular interest in skeletal reconstructions is the nature of the vertebral column and its component vertebrae including the relative lengths, widths and inclination of the vertebral processes: the neural spine, the transverse processes, the pre- and post-zygapophyses. In mammals these structures are the sites of origin and insertion of the muscle tendon systems (*m*) that both stabilise and flex the vertebral column including the *m*. *transversospinalis*, *m*. *multifidus lumborum* and *mm*. *rotatores* muscles that, along with the *m*. *biceps femoris*, *m*. *caudofemoralis* and *m*. *adductor cruris* muscles, correlate well with hindlimb movement and the locomotory mode of a species, being indicators of both posture and gait in mammals [17, 18, 19, 20, 21].

The caudal skeletons of all the marsupials we studied, living and extinct, are less revealing than the trunk vertebrae, differing only in overall proportions and in the number of distal vertebrae. The muscles controlling the tails of the extant species, the flexors and extensors, have multiple origins including the articulating processes of the last thoracic vertebra, the lumbar vertebrae, sacrum, pelvis and the proximal caudals [22, 23]. Their tendons insert on the lateral and mammillary processes of the caudal vertebrae. However, the proximal caudal vertebrae also serve as sites of origin of the caudal head of the hind limb flexors and the abductors of the hip and lateral flexors of the tail [23, 24 and refs. therein]. The relative surface area and length of these lateral caudal processes, mammillary bodies and chevrons are indicative of the strength (cross-sectional area) and mobility (lever arm of lateral processes) of the tail [18]. Accordingly, to infer tail use in *T*. *carnifex* we have made structural comparisons with extant forms in which tail use is known.

The primary aim of this paper is to describe hitherto missing elements in the skeleton of *Thylacoleo carnifex* and, by comparison with extant marsupials and previously published material, to integrate this information into a reappraisal of postcranial biomechanics and anatomy of this enigmatic species.

## Materials and methods

Comparative vertebral measurements of extant marsupial species were made on both complete articulated and disarticulated skeletons of individual adult animals drawn from collections housed in the Flinders University Palaeontology Laboratory (FUR), Western Australian Museum (WAM), the South Australian Museum (SAMA P, SAMA M) and the Tasmanian Museum (TMAG A) as follows: *Thylacinus cynocephalus* (*Thy*. *cynocephalus*) TMAG A300, A315♂, A312 f, SAMA M95 f, M665 f, M1959, M1960; *Sarcophilus harrisii* (*S*. *harrisii*) FUR012, TMAG A3505, A303 juv., SAMA M1994, M18397; *Dasyurus viverrinus* (*D*. *viverrinus*) SAMA M673, M7222, FUR027; *Trichosurus vulpecula* (*Tri*. *vulpecula*) FUR036, FUR030, SAMA M669 ♂, SAMA M18397; *Phascolarctos cinereus* (*P*. *cinereus*) FUR009, FUR003; *Lasiorhinus latifrons* (*L*. *latifrons*) FUR006, SAMA M5986; *Macropus fuliginosus* (*M*. *fuliginosus*) M667♂; *Macropus rufus* (*M*. *rufus*) FUR 001♂. Raw data is presented in S1 Table.

The presence of articulated skeletal material required particular care in excavation. The Naracoorte *Thylacoleo carnifex* (*T*. *carnifex*) specimens, from Komatsu Cave (5U240) in Henschke’s Quarry at Naracoorte, when found, were partially buried in a soft, powdery grey silt and sand. They were photographed, hardened and excavated as described by Wells et al. [9]. They comprised the disarticulated and scattered remains of 11 individuals including 2 partially articulated skeletons (SAMA P43220, P43221).

The Nullarbor *T*. *carnifex* specimens from Flight Star Cave, Last Tree Cave and Leaena’s Breath Cave had been treated in a similar fashion, photographed, hardened and removed to the Western Australian Museum as described by Prideaux et al. [10]. They included WAM 02.7.1, a complete skeleton that was found isolated and apparently undisturbed where it had lain, likely at least since the middle Pleistocene [10], as well as five partial and disarticulated skeletons viz: WAM 02.7.2; WAM 02.7.3; WAM 02.7.6; WAM 02.7.7; and WAM 02.7.8.

We confined our analyses to those individuals where we were confident of the element associations. Thoracic vertebrae were identified by the presence of demi-facets on the proximal and distal lateral aspect of the centrum. Lumbar vertebrae were identified by the absence of diapophyses for rib-head articulations, presence of a neural spine and transverse processes. An intervertebral spacing of 2–3 mm was allowed in reconstructions of the vertebral column. This spacing approximated that recorded in the partially-buried articulated specimens prior to excavation. All skeletal elements were measured using digital calipers (Mitutoyo). As sexual dimorphism in the forelimb has been documented for large macropodids [25,26] we have indicated sex where known for all extant specimens.

The articulated *T*. *carnifex* material from Komatsu Cave, Henschke’s Quarry, Naracoorte, was used as a template to reassemble the disarticulated Nullarbor specimens. All vertebrae, cervical to transitional caudals (prezygopophyses, vestigial postzygopophyses), from the Nullarbor specimen WAM 02.7.2 were threaded onto a brass rod passing through the neural canal. Pads of modelling clay 2–3 mm thick were placed between vertebrae to represent disc spacing. The brass rod was bent to allow the pre- and post-zygapophyses to align in a comfortable non-flexed position with centrum endplates parallel. This resulted in the lateral processes aligning. Assembled specimens were photographed in lateral and dorsal aspect. Anatomical texts and published papers were consulted for identification of muscle origins and tendon insertions (viz. placental mammals—the dog [27]; the cat [28]; prehensile and non-prehensile tails in primates- [29, 30, 31, 32] marsupials–[23, 24, 33, 34, 35]). The following indices based on these vertebral measurements were used to compare the skeleton of *T*. *carnifex* to a range of extant marsupials in which some or all aspects of posture, gait, tail structure and function are known. Not all parameters for all vertebrae could be measured for all species studied as some specimens were either damaged or some very distal caudal vertebrae were missing.

1. vertebral robusticity index (RI) = transverse diameter of anterior facet of centrum/centrum length, where minimum diameter is measured approximately midway between centrum end plates while length is measured as overall length between endplates. Large values, broad and short = robust, small values, narrow and long = slender.
2. vertebral resistance to bending in the sagittal plane (RSB) = bh^2^/100 where b = proximal width and h = proximal height of centrum [17].
3. vertebral resistance to bending in the lateral plane (RTB) = hb^2^/100 [17]
4. bending resistance = RSB/centrum length and RTB/centrum length; this is a modification of bending resistance where we have also taken the length of the centra into account. It is designed to reflect the resistance to bending more accurately than simply looking at the proportions of the articular surface of the centrum (modified from Slijper [17] and Youlatos [31]).
5. RSB vs. RTB- expressed as a ratio. This is designed to illustrate the relative resistance to sagittal and transverse stresses along the vertebral column. A high value is indicative of proportionally high resistance to sagittal bending, in turn suggesting that in life that part of the vertebral column was exposed to higher sagittal stresses.
6. relative expansion of the Transverse Process Index (TPI) = (maximum transverse width/vertebral centrum length)*100, modified from Youlatos [31] who used the *proximal* width of the caudal vertebrae. However, this measurement is essentially meaningless whereas the *maximum* dimensions of the transverse processes will provide an indication of the expansion of the attachments of the main rotator and flexors of the lumbar spine and tail. Higher values indicate more expanded muscular attachments and accordingly more powerful muscles [29, 30].
7. results from methods 1–3 and 5 were also log transformed in order to emphasise differences between taxa.
8. Tail taper, diameter to length of each caudal vertebra, was calculated as (Cd 1 prox. centrum dia.- Cdn)/∑(Cd1 –Cdn length).
9. Sacral taper was calculated as (maximum width of most posterior sacrum/max. W of most anterior sacrum)/length of sacrum.

In addition we compared the zygapophyseal joint profiles to the six categories defined by Boszczyk et al. [20]. Of particular value to this study was the excellent atlas of whole body x-rays of anaesthetised extant marsupials by Vogelnest & Allan [11]. These provided a more accurate gauging of intervertebral spacing as well as a clear view of vertebral column flexure in the living animals. The form revealed in lateral x-ray views stands in marked contrast to that of a number of reassembled museum skeletons.

We also revisited previously published descriptions [4, 5, 6] of those aspects of the axial and appendicular skeleton of *T*. *carnifex* that relate to locomotion.

Terminology, where applicable, is based on the Nomina Anatomica Veterinaria [36].

## Results

### The Skeletons

#### (i) Nullarbor skeletons

**Fig 1:**
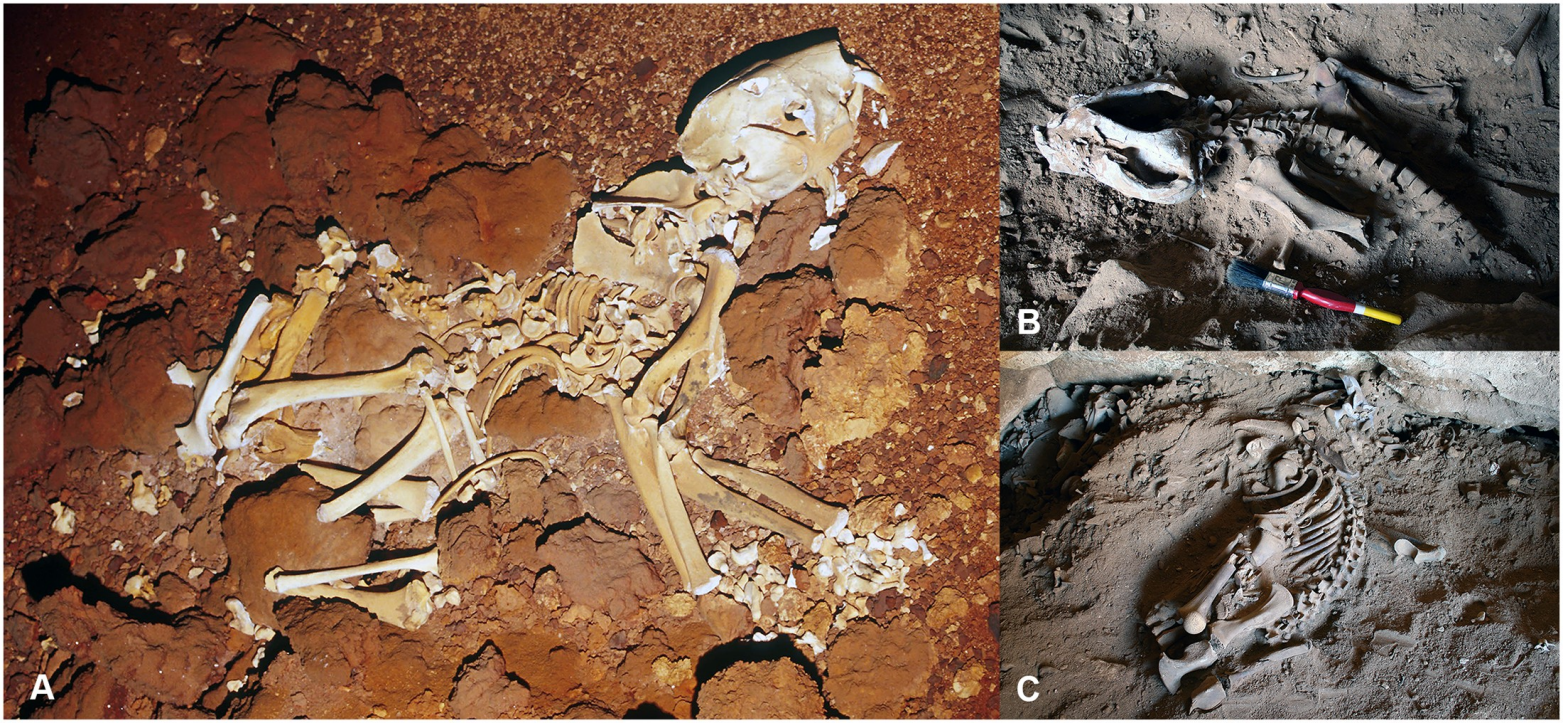
*Thylacoleo carnifex* skeletons. (A) Complete and isolated skeleton of *T*. *carnifex* (WAM 02.7.1) as found in Flight Star Cave, Nullarbor Plain, Western Australia. Individual bones remain associated with some limb elements still in articulation and caudal skeleton curved dorsally above the pelvis (Photo: Clay Bryce); partially articulated skeletons of *T*. *carnifex* (B) SAMA P43220 (KC11) and (C) SAMA P43221 (KC4) as uncovered in Komatsu Cave, Henschke’s Quarry, Naracoorte, South Australia. (Photos: S. Bourne).

The Nullarbor skeleton (WAM 02.7.1) is illustrated in Fig 1A. Although not articulated, all vertebrae with the exception of the caudals remain in close association. The more widely separated caudal vertebrae appear in correct anatomical sequence in an arc around the pelvis and dorsum.

#### (ii) Naracoorte skeletons

Remains of eleven individuals were uncovered in Komatsu Cave, Henschke’s Quarry, Naracoorte (Field numbers KC 1–11). The two most complete specimens used in this study (KC 4 and 11), are registered as SAM P43221 and P43220 (Fig 1B and 1C). They include articulated thoracic, lumbar and sacral vertebrae and crania with limb bones and some adjacent caudal vertebrae.

The preservation of articulation and posture in the vertebral column of P43221 shows no obvious evidence of distortion from tendon shortening. This individual was used as a reference when articulating the vertebrae of other specimens.

### Caudal skeleton: Description and comparison

**Fig 2:**
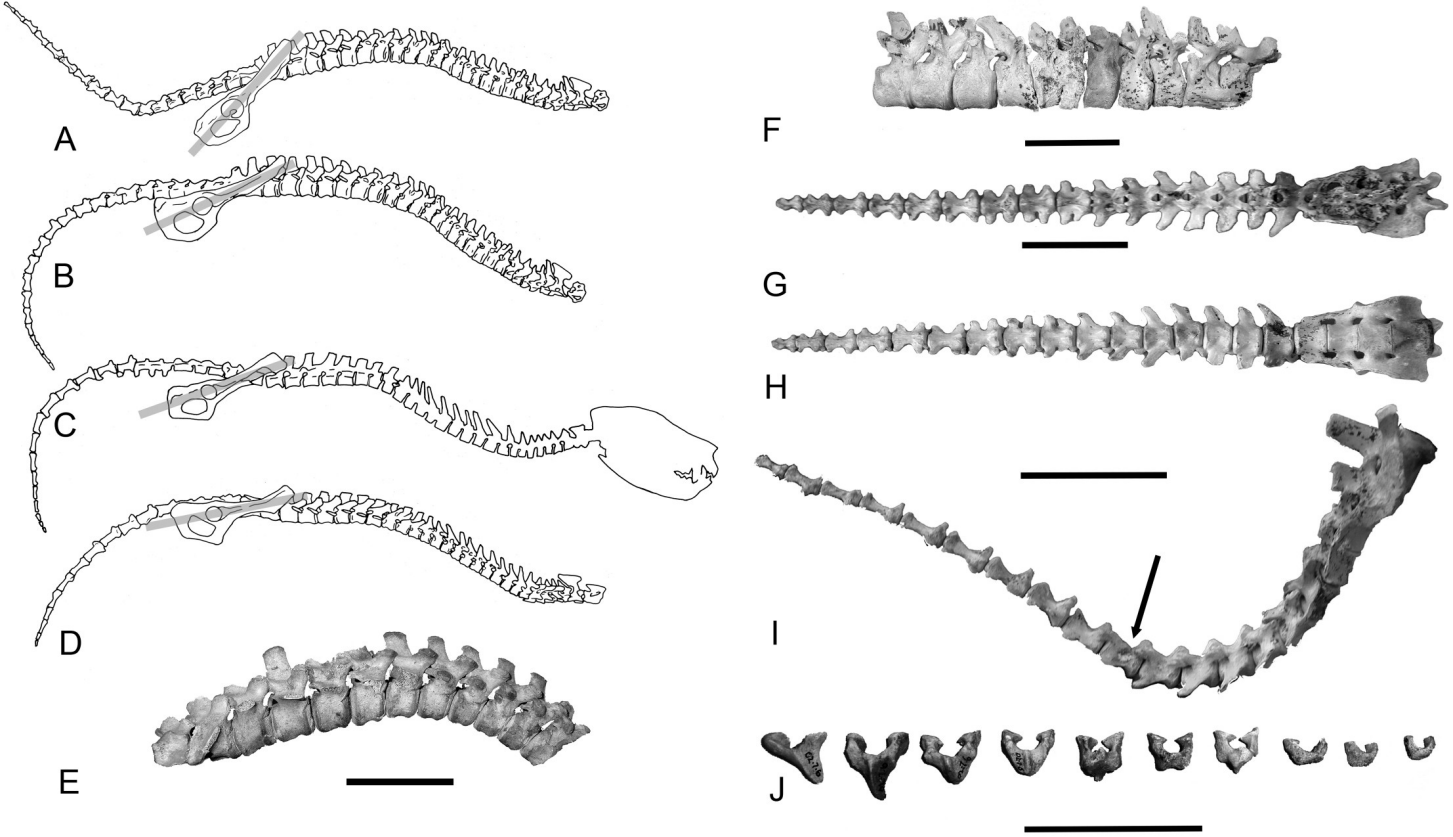
Aspects of the axial skeletons of *T*. *carnifex* and *S*. *harrisii*. (A-D) Comparisons between the vertebral columns of *T*. *carnifex* and *S*. *harrisii*. (A) Assembled vertebral column of *T*. *carnifex* (WAM 02.7.2) with tail in natural position based on orientation of articular facets; (B) with tail ventrally flexed; (C) compared with the nearest extant morphological analogue *S*. *harrisii* traced from x-ray [11]; (D) *S*. *harrisii* vertebral column from Tasmanian Museum mounted skeleton (grey bars represent the orientation of the pelvis relative to the vertebral column, A-D have all been scaled to the same size for comparative purposes, note the dorso-ventral flexion of the cervical-thoracic column and rigid lumbar, sacral and proximal caudal region); (E) articulated thoraco-lumbar region of *T*. *carnifex* as preserved in SAMA P43221 (scale bar equals 50 mm); (F) pathologies on thoracic vertebrae 1/2, 6/7 & 8 of *T*. *carnifex* WAM 02.7.2 (scale bar equals 50 mm, note the fusion of the of T1/2 centra possibly the result of calcification of tendons, erosional damage at interface of T6/7 and lipping of the ventral anterior aspect of T8); the tail of *T*. *carnifex* assembled (WAM 02.7.2) (G) ventral view, (H) dorsal view (the sacrum comprising four fused vertebrae is followed by three forms of caudal vertebrae (i) proximal nos. 1–6 (ii) transitional no. 7 (iii) distal nos. 8–16., remaining 5 distal caudals in the series omitted, (scale bar equals 100 mm); (I) the tail of *T*. *carnifex* showing maximum extent of dorsal flexion in proximal caudal vertebrae (arrow indicates transitional vertebra caudal 7[, scale bar equals 100 mm); and (J) chevron bones collected in the vicinity of the tail of WAM 02.7.1 (scale bar equals 100 mm).

The caudal skeleton assembled from the Nullarbor specimen (WAM 02.7.2) is illustrated in Fig 2A, 2B and 2G–2I. The caudal vertebrae can be divided into three morphotypes (Fig 2G–2I) viz.

1. proximal vertebrae (Cd1–6), all bearing pre- and post-zygapophyses as well as single rear-swept transverse processes;
2. a transitional caudal vertebra (Cd7), distinguished by the presence of articulating prezygapophyses and vestigial non-articulating postzygapophyses;
3. distal vertebrae (Cd8–20), distinguished by the absence of zygapophyses and with reduced or absent pedicle remnants of the neural arch.

In tailed mammals the transverse processes of the proximal caudals serve both as sites of origin and insertion of the caudal musculature, as well as sites of origin for abductors of the thigh (*m*. *caudofemoralis* caudal head). In *T*. *carnifex* the transverse processes on Cd1–7 are broad and strap-like and deflected posteriorly, those of Cd1–5 are of approximately equal length, those of Cd6–7 progressively shorter. Cd8–9 retain the last vestiges of the lateral processes which are bifurcate. A small smooth round notch occurs at the base of the two arms. All subsequent caudal vertebrae from Cd11 to the tip of the tail exhibit a dumbbell form (Fig 2G and 2H).

**Fig 3:**
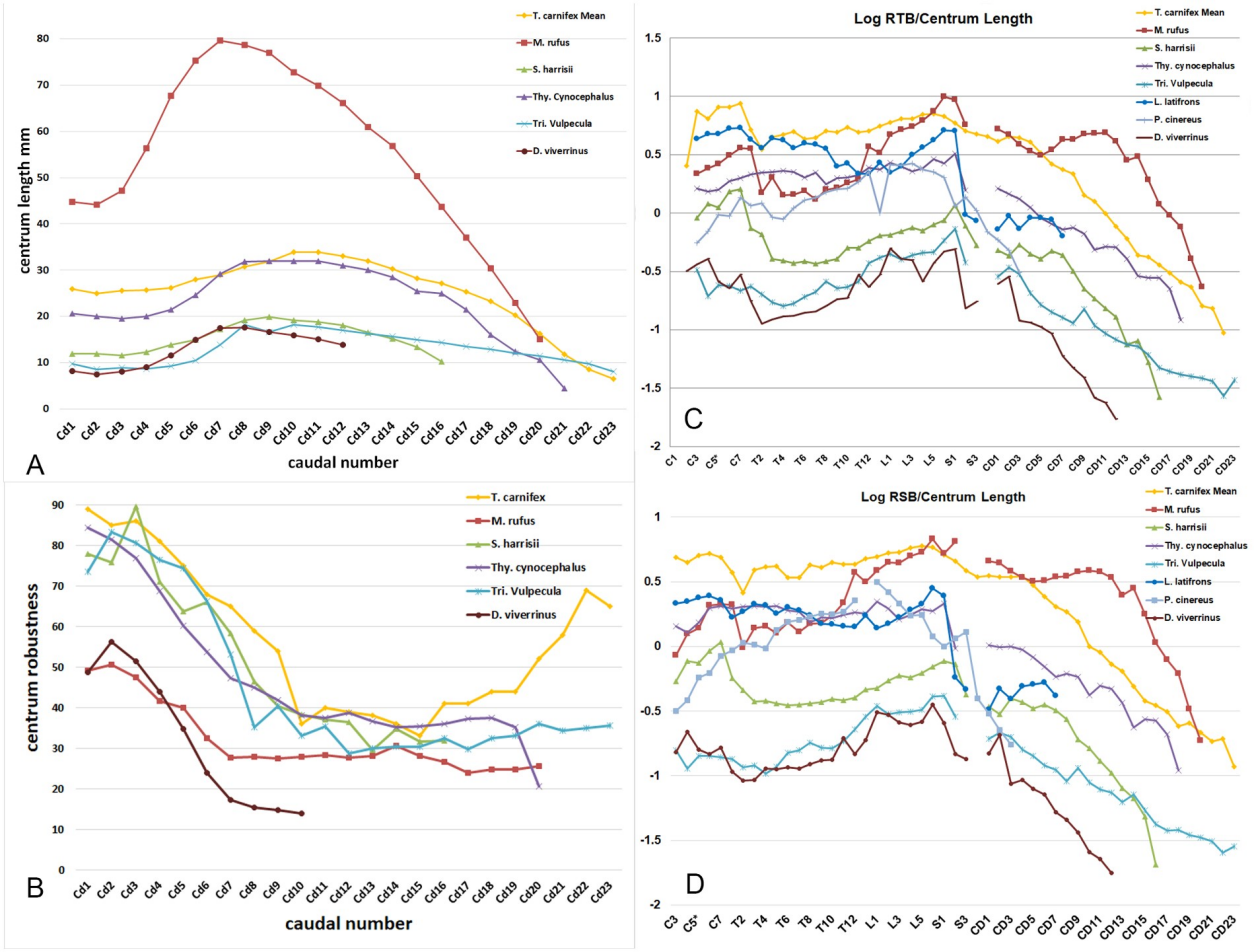
Caudal robusticity and bending resistance in the axial skeleton. (A) Centrum length for caudal vertebrae of a selection of arboreal, scansorial and saltatorial marsupial species and *T*. *carnifex* (mean); (B) robusticity of caudal vertebral centra of the same group of marsupials; (C) log transformed RSB relative to centrum length; and (D) RTB relative to centrum length. Plots C and D include the total vertebral column (except for the axis and atlas vertebrae) for *T*. *carnifex* and the other marsupials studied. Gaps in the curve relate to variation in the number of sacral vertebrae present.

Caudal centra increase in length in sequence from the base of the tail to Cd10–11 then decrease to the tip of the tail (Fig 3A). Pre- and post-zygapophyses are confined to Cd1–7. A similar pattern of change in centrum length was found in all other tailed marsupials studied, with the peak in length around Cd7–9 (Fig 3A). The diameter-to-length ratios of the caudal centra provide a measure of vertebral proportions i.e. high values = short and broad (robust), low values = long and narrow (slender). Vertebral proportions for tails of all studied taxa except the vestigial-tailed *Phascolarctos cinereus* and *Lasiorhinus latifrons* are plotted in Fig 3B. Most tailed species studied show a similar pattern with a gradual increase in vertebral centra proportions to the transitional vertebra followed by a gradual decline to the tip of the tail. The caudal vertebrae of *T*. *carnifex* and to a lesser extent *Trichosurus vulpecula* differ from this pattern with an increase in robusticity associated with a foreshortening of the distal caudal vertebrae (Fig 3B).

Having established patterns of proportional change along the caudal vertebrae for all species irrespective of tail size, we calculated the RSB and RTB for the whole vertebral column (Fig 3C and 3D). The caudal pattern in *T*. *carnifex* is similar to that of all other species studied, differing only in the absence of a secondary peak in RSB around the region of the transitional caudal vertebrae (C7). The RTB follows a similar pattern (Fig 3D).

The rate of taper in the tail of *T*. *carnifex* (2.9%) is most similar to that of *Thylacinus cynocephalus* (3%) and *Sarcophilus harrisii* (2.7%) and more than *Macropus rufus* (2%), *Tri*. *vulpecula* (1.4%), *Dasyurus viverrinus* (1.4%). Species with lower taper rates are those with proportionally steeper decline in bending resistance distally, those species with higher rates of taper have proportionally greater resistance to bending.

The tail of WAM 02.7.1 also included nine chevron bones (Fig 2J) of which the smallest remained attached to the distal end of Cd13. They have been arranged according to size and keel configuration resembling that found in species of *Macropus*. If this order is correct then these chevrons would come to lie along the ventral surface of the tail from around the Cd5–6 junction through to the Cd13–14 junction, the region of maximum centrum length (Fig 3A and 3B). In mammals chevrons serve to both shield the ventral caudal vasculature and also to anchor the tendons of the *m*. *flexor caudae lateralis* and *m*. *flexor caudae medialis* [18, 37]. The chevrons of *T*. *carnifex*, in marked contrast to those of *M*. *rufus*, are weakly keeled.

### Axial skeleton: Description and comparison

The total number of vertebrae preserved for each specimen of *T*. *carnifex* and their position in the axial skeleton is indicated in Table 1. The number of fused sacral vertebrae increased ontogenetically, additions occurring at the caudal end in sequence (Cd1 becomes S2, Cd2 becomes S3 and Cd3 becomes S4). Although a juvenile tail is not currently known, this would imply that in juveniles there are an extra three proximal caudal vertebrae and that the transitional vertebra would be Cd10. This may have implications for an ontogenetic change in tail function. The maximum number of vertebrae in adult specimens comprised 7 cervical, 13 thoracic, 6 lumbar, 4 sacral and 23 caudals.

**Table 1:** Vertebral columns of five *T*. *carnifex* specimens used in this study including total number of vertebrae along with positional data for each vertebral class (e.g. cervicals 1,2,3,4,5,6,7 are indicated as 1–7).

*Thylacoleo carnifex* vertebrae							
	WAM 02.7.1	WAM 02.7.2	WAM 02.7.6		SAMA P43221		SAMA P43220
Total preserved	52	46	52		28		22
Cervical	1–7	1–7	1–7		1–7		-
Thoracic	1–13	1–13	1–13		1–13		7–13
Lumbar	1–6	1–6	1–6		1–6		1–6
Sacral	1–3	1–4	1–3		1–2		1–2
Caudal	1–23	1–16	1–23		-		1–3
Vertebrae of other taxa studied							
	*Sarcophilus harrisii*	*Phascolarctos cinereus*	*Thylacinus cynocephalus*	*Macropus rufus*	*Lasiorhinus latifrons*	*Dasyurus viverrinus*	*Trichosurus vulpecula*
Cervical	7	7	7	7	7	7	7
Thoracic	13	11	13	13	13	13	13
Lumbar	6	8	6	6	6	6	6
Sacral	3	3	2	2	4	3	3
Caudal	19	7	21	20	15	22	23

At the time of investigation the most complete vertebral column, WAM 02.7.1, remained on display at the Western Australian Museum with all elements arranged in anatomical sequence, and was not available to be re-assembled as a standing skeleton. Accordingly we re-assembled the near complete vertebral column of WAM 02.7.2 (Fig 2A and 2B) allowing for intervertebral disc spacing. In doing so, the spine assumed a curvature similar to that of the Naracoorte specimen P43220 (Figs 1B and 2E). We also re-assembled several partial vertebral columns from other Naracoorte and Nullarbor specimens. Whether juvenile or adult, all exhibited a similar curvature in both the sagittally-flexed and extended positions. Spinal curvature of the re-assembled *T*. *carnifex* showed the greatest similarity to lateral whole body x-rays of extant, quadrupedal, dasyurid marsupials, *S*. *harrisii*, *D*. *viverrinus* and *Phascogale calura* [11]. Of particular note was the ventral flexure of the cervical–thoracic column followed by a relatively rigid and straight lumbar–sacral–proximal caudal sequence along with a sharp upward bend in the transitional caudals (Fig 2A).

**Fig 4:**
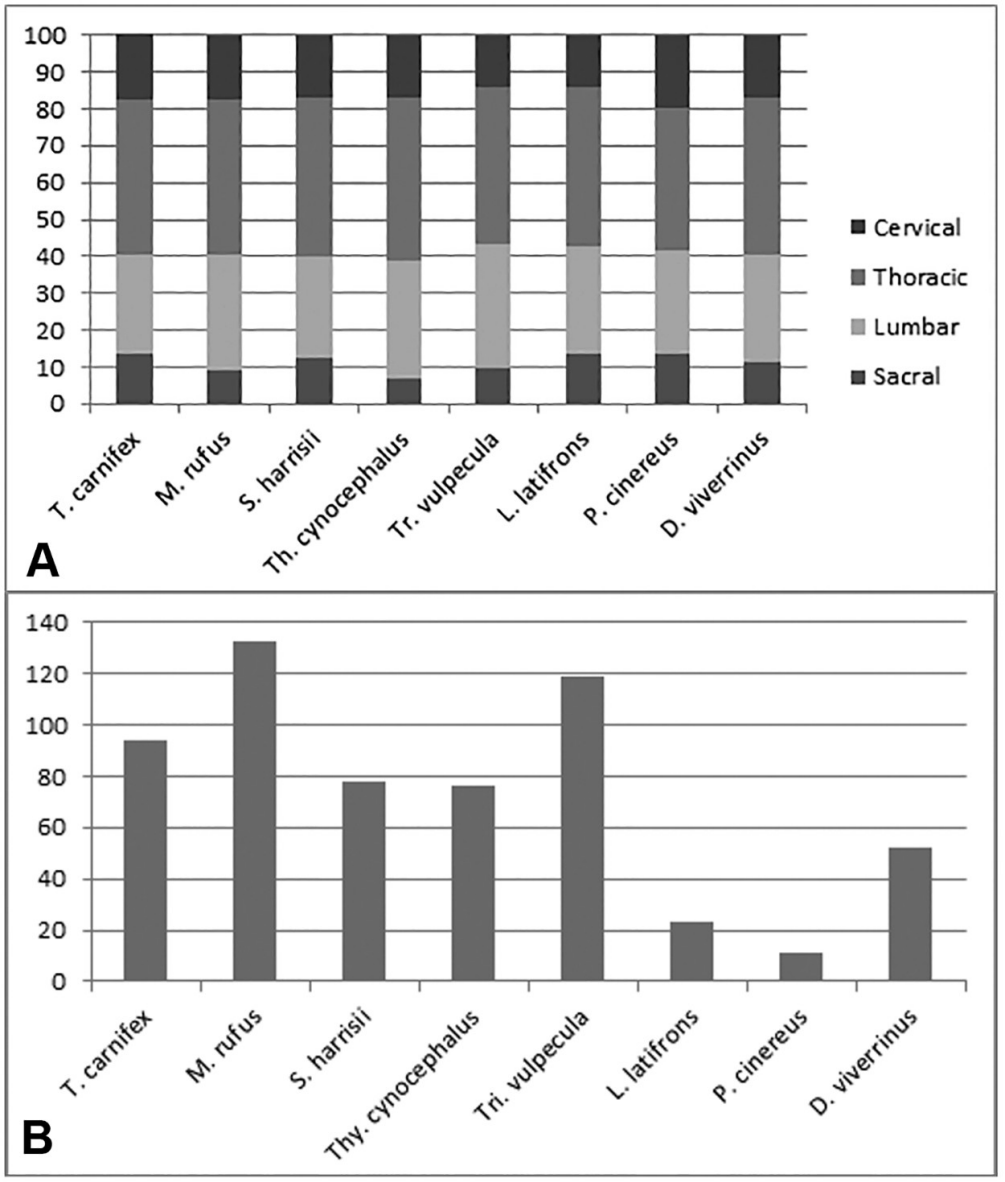
Vertebral column proportions. (A) Proportions of different segments of the complete marsupial vertebral column expressed as percentage of vertebral column length (excluding caudals); (B) Tail length expressed as a percentage of the length of the rest of the axial skeleton. *T*. *carnifex* is compared with adult arboreal /scansorial (*Tri*. *vulpecula*, *P*. *cinereus*, *D*. *viverrinus*), scansorial/terrestrial (*S*. *harrisii*), cursorial (*Thy*. *cynocephalus*), fossorial (*L*. *latifrons*) and saltatorial (*M*. *rufus*) species.

The *T*. *carnifex* axial columnar proportions are compared with those of quadrupedal marsupial carnivores, *Thy*. *cynocephalus*, *S*. *harrisii*, *D*. *viverrinus* and marsupial herbivores, *Tri*. *vulpecula*, *L*. *latifrons*, *P*. *cinereus* and bipedal *M*. *rufus* in Fig 4A. Proportions are expressed as a percentage of total column length (CL) excluding caudal vertebrae. The single sample for each of the comparative specimens reflects the rarity of totally complete yet disarticulated individual skeletons within the museum collections we studied. Accordingly, we justify the use of single specimen comparisons following Finch & Freedman [5] who noted very little difference in proportions of a large sample of *Tri*. *vulpecula*.

Tail length expressed as a percentage of combined length of the remainder of the vertebral column (CL) is shown in Fig 4B. The most complete tail (WAM 02.7.1) comprised almost half (49%) the overall vertebral column length. When expressed as a percentage of CL, the tail of *T*. *carnifex* (138%) is longer than *S*. *harrisii* (110%) and shorter than *Tri*. *vulpecula* (158%) and *M*. *rufus* (180%) (Fig 4B). The only bipedal species examined, *M*. *rufus*, had the longest tail relative to column length.

**Fig 5:**
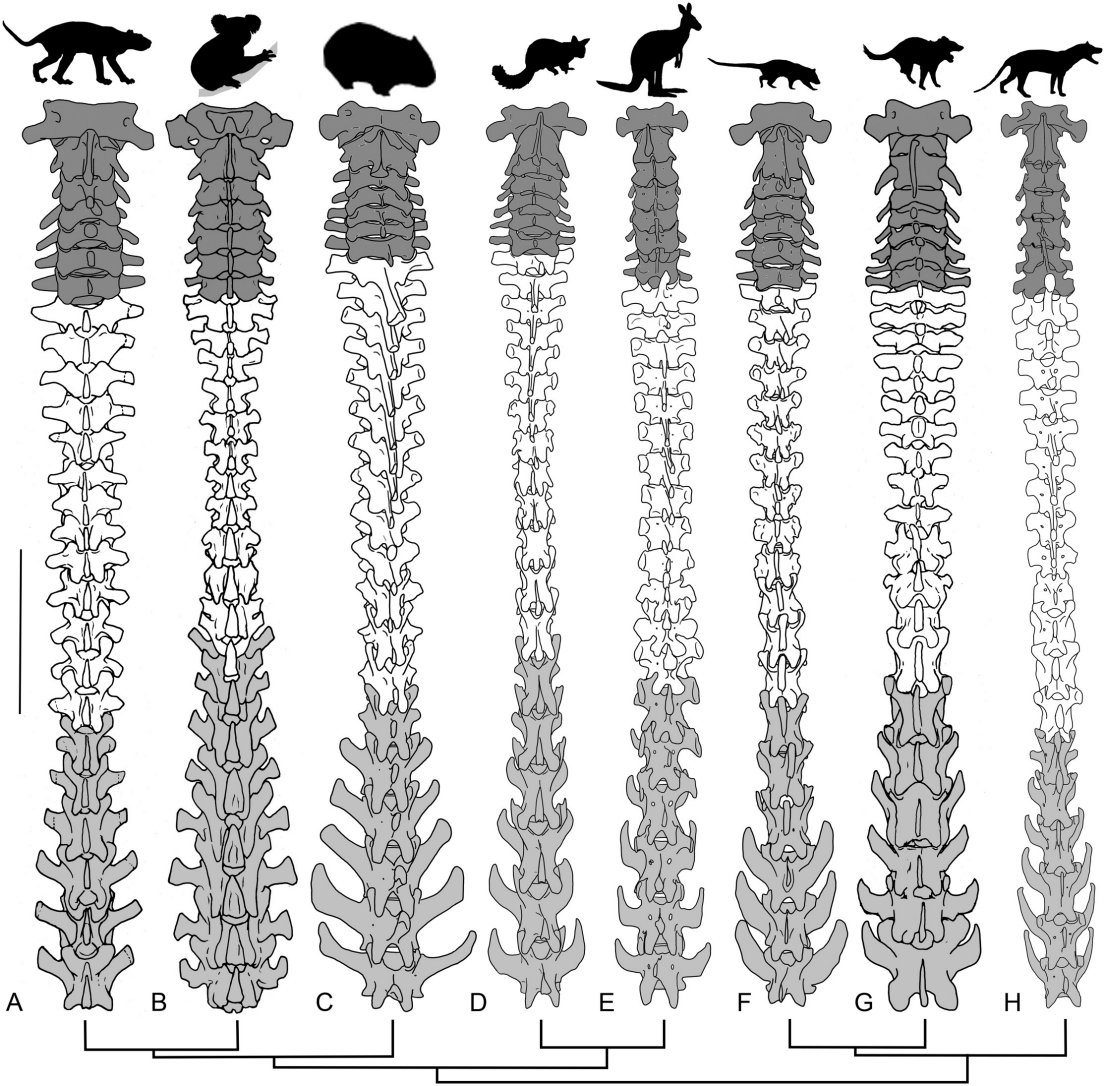
Vertebral columns of the study species excluding the sacral and caudal vertebrae. Dorsal view of the cervical (dark grey), thoracic (white) and lumbar (light grey) vertebral columns of (A) *T*. *carnifex*; (B) *P*. *cinereus*; (C) *L*. *latifrons*; (D) *Tri*. *vulpecula*; (E) *M*. *rufus*; (F) *D*. *viverrinus*; (G) *S*. *harrisii*; and (H) *Thy*. *cynocephalus*. Scale bar equals 100 mm for *T*. *carnifex*, other taxa are scaled for proportional comparison. Phylogenetic relationships displayed at bottom of figure are based on [41].

*Thylacoleo carnifex* has a relatively long neck (17.8% CL, see also Fig 5); a feature it shares with the carnivores *S*. *harrisii* and *D*. *viverrinus* (17%) and *M*. *rufus* (17.3%), but not as long as *P*. *cinereus* (19.9%). The sacral length in adult *T*. *carnifex* is proportionally similar to that of *P*. *cinereus* and *L*. *latifrons* at around 19% of trunk length. There may be two, three or four, fused sacral vertebrae, the number increasing with the relative age of the individual as evidenced by the degree of epiphyseal fusion. Nonetheless, with the exception of the bipedally-hopping *M*. *rufus*, the combined lumbar sacral length as a proportion of trunk length is similar in all species. The extended sacral region in both *T*. *carnifex* and *S*. *harrisii* is associated with a relatively longer sacro-iliac joint.

The lower back of *T*. *carnifex* comprises six lumbar vertebrae. The vertebral bodies are robust, increasing in cross-sectional area and width caudally. The end-plates are largely parallel with only a very slight convergence ventrally. In lateral view the caudal faces of the centra are progressively offset ventrally from the cranial faces to produce, when assembled, a gradual postero-ventral cant to the lumbar column (Fig 2E).

The robusticity index (minimum centrum diameter/length) for the lumbar vertebrae of *T*. *carnifex* ranked highest among the marsupials we examined (rank order as follows: *T*. *carnifex; S*. *harrisii; P*. *cinereus; Thy*. *cynocephalus; M*. *rufus; L*. *latifrons; D*. *viverrinus; Tri*. *vulpecula* (Table 2)).

**Table 2:** Average robusticity index (RI) of adult lumbar vertebrae expressed as means ± standard deviation, arranged in rank order (n = 1 unless otherwise indicated).

*Thylacoleo carnifex* (adults n = 4)	83.58 ±4.37; 80.9±3.7; 82.2±4.5; 97.9±3.5
*Sarcophilus harrisii*	76.51±3.12
*Phascolarctos cinereus*	72.98±11.28
*Thylacinus cynocephalus*	66.78±3.68
*Macropus rufus*	58.16±2.97
*Lasiorhinus latifrons*	58.12±3.69
*Dasyurus viverrinus*	50.74±7.91
*Trichosurus vulpecula*	42.51±2.54

**Fig 6:**
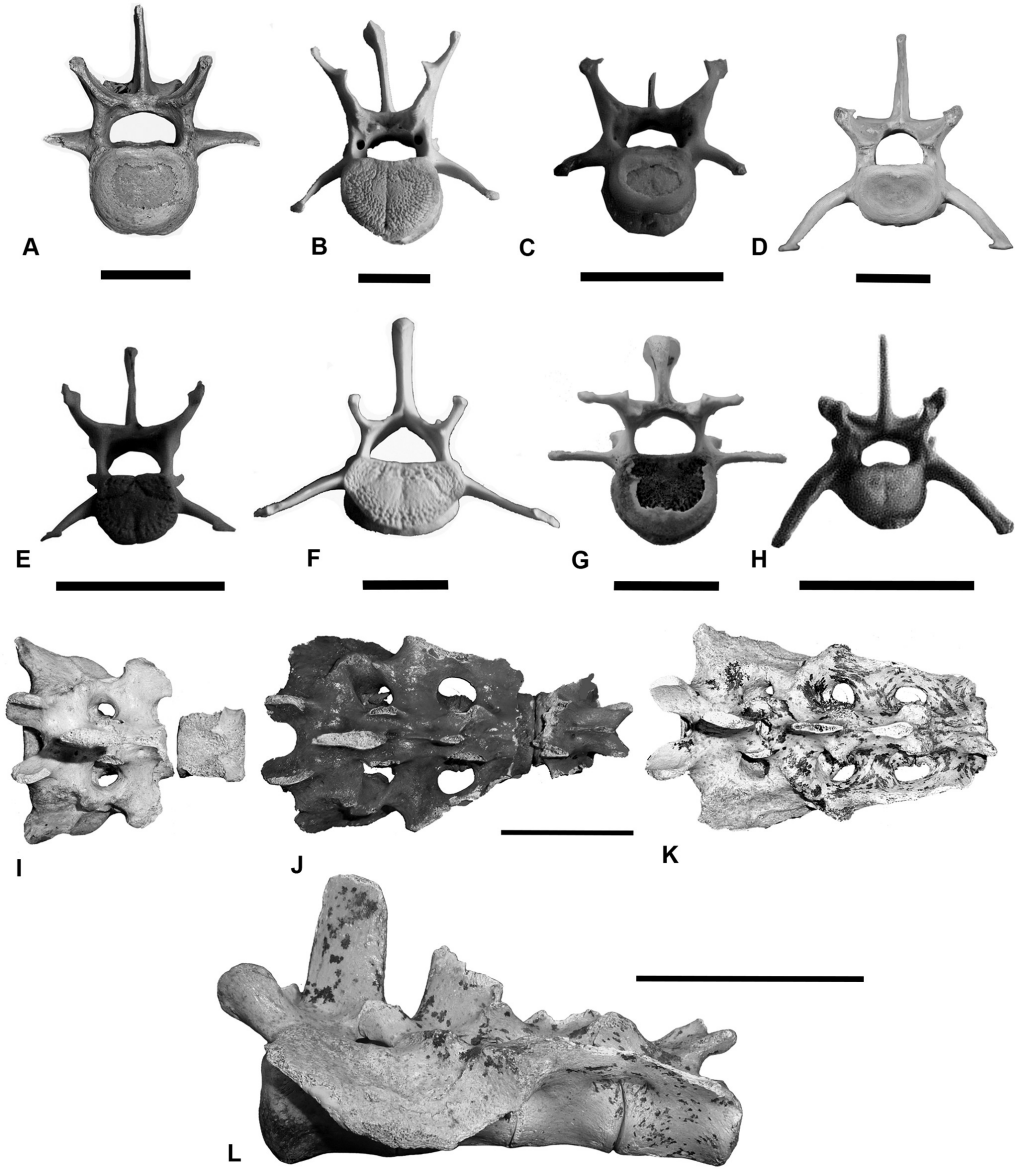
Lumbar vertebrae of the study species and sacrum of *T*. *carnifex*. Cranial view lumbar vertebra four (L4), (A) *T*. *carnifex*; (B) *M*. *rufus*; (C) *S*. *harrisii*; (D) *Thy*. *cynocephalus*; (E) *Tri*. *vulpecula*; (F) *L*. *latifrons*; (G) *P*. *cinereus*; (H) *D*. *viverrinus*; (I-K) growth stages in the sacrum of *T*. *carnifex* (I) two, (J) three, (K) four fused vertebrae; and (L) lateral view of (K) showing deep pocketing of articulation with ilium. Note the similarity in form between (A) & (C) saddle-shaped prezygapophyses, short neural spine and lateral processes, scale bars in A-H equal 20 mm. I-K all to same scale, both sacral scale bars equal 50 mm.

In *T*. *carnifex*, the pre- and post- zygapophyses that link the vertebral bodies are widely spaced, broad, U-shaped and positioned centrally on tall, robust pedicles. They are most similar in form to those of *S*. *harrisii* and *P*. *cinereus* (Fig 6).

In all mammals the inclination of the spinous processes tends to be at right angles to the line of action of the muscles and ligaments attached to them while their relative length is proportional to the forces acting upon them [17]. In *T*. *carnifex* the neural spines of the lumbar and sacral vertebrae are upright or inclined slightly caudally relative to the axis of the centrum, the inclination decreasing towards the sacrum (Fig 2A and 2E). This upright to caudal inclination in the post diaphragmatic vertebrae along with the relative shortness of the vertebral centra are features associated with a reduction in mobility and enhancement of tension musculature in the lower back. It is a feature of animals in which the lower back is flat or dorsally arched.[17]. The shape and inclination of the spines in *T*. *carnifex* were similar to *S*. *harrisii*, *P*. *cinereus*, *Tri*. *vulpecula*, and *M*. *rufus* rather than a cranial inclination as in *D*. *viverrinus*, *Thy*. *cynocephalus* and *L*. *latifrons*.

In *T*. *carnifex* the transverse processes of the lumbar vertebrae are relatively short and broad (Figs 5 and 6A). They arise close to the base of the pedicles projecting at right angles to the centrum with both a forward cant and only a slight ventral deflection. There is no forward overlap with adjacent vertebrae. This condition is similar to that of *S*. *harrisii* and *P*. *cinereus* and distinct from the marked forward angling and overlap exhibited in all the other species studied. The transverse processes increase in length and width from L1 to L6 (Fig 5). There is no reduction in transverse length where they pass between the ilial blades of the pelvis.

The RSB and RTB for each centrum along the total vertebral column both for *T*. *carnifex* and a selection of marsupials is plotted in Fig 3C and 3D. The values for the five adult *T*. *carnifex* are shown as a mean. The peak RSB in *T*. *carnifex* occurs around the mid-lumbar region with two lesser peaks in the cervical and the mid-proximal caudal vertebrae. The lowest resistance to sagittal flexion occurs in the anterior and mid-thoracic region. On this point, it is of interest to note the fusion of the T1–2 centra (possibly the result of calcification of tendons), erosional damage at interface of T6–7 and lipping of the ventral anterior aspect of T8 in one of the *T*. *carnifex* specimens (WAM 02.7.2), features suggestive of repetitive strain damage in life (Fig 2F).

The log-transformed pattern of resistance to both lateral and sagittal centrum bending was similar in the trunk vertebrae of all species studied with peaks occurring around the cervical–thoracic, lumbar–sacral junctions (Fig 3C and 3D). Species differences were largely a matter of magnitude. Vertebral columns exhibiting the overall greatest resistance to bending were in rank order, strongest to weakest: *M*. *rufus*, *T*. *carnifex*, *Thy*. *cynocephalus*, *L*. *latifrons*, *P*. *cinereus*, *S*. *harrisii*, *Tri*. *vulpecula* and *D*. *viverrinus*. All tailed species, with the exception of *T*. *carnifex*, *Thy*. *cynocephalus* and *S*. *harrisii* exhibit a similar pattern of resistance in the proximal caudal vertebrae, declining from the sacral junction rather than from the transitional caudal. Resistance in the proximal caudals varies between species and from Cd1 to the transitional caudal. In *T*. *carnifex* it peaks around Cd3–4 and then begins to decline; the opposite of that seen in most other species studied. The distal caudals in all species exhibit a rapid and uniform decline in bending resistance to the tip of the tail, with the exception of *Tri*. *vulpecula*, in which the decline is more gradual (Fig 3A–3D). Among all the species used in this study the resistance curves for *T*. *carnifex*, *S*. *harrisii*, *M*. *rufus* and *Thy*. *cynocephalus* show the greatest similarity in pattern with resistance peaks around the shoulder (C7-T1) and hip (L6-S1) region of the column. The pattern is typical of terrestrial quadrupeds in which a greater part of the sagittal stress is exerted by the hind limbs and a lesser amount by the forelimbs. In contrast to the four taxa aforementioned, the major RSB in the vertebral columns of the arboreal *P*. *cinereus* and scansorial *Tri*. *vulpecula* and *D*. *viverrinus* is confined to the lumbar–sacral area, indicative of the loading by the hind limbs when climbing, but no peak around the forelimbs. It is likely that *M*. *rufus* displays a peak around the cervical–thoracic junction due to a combination of limb use during pentapedal locomotion (where a large portion of the body mass rests solely on the forelimbs) and intraspecific confrontations (the forelimbs being important in male-male encounters).

**Fig 7:**
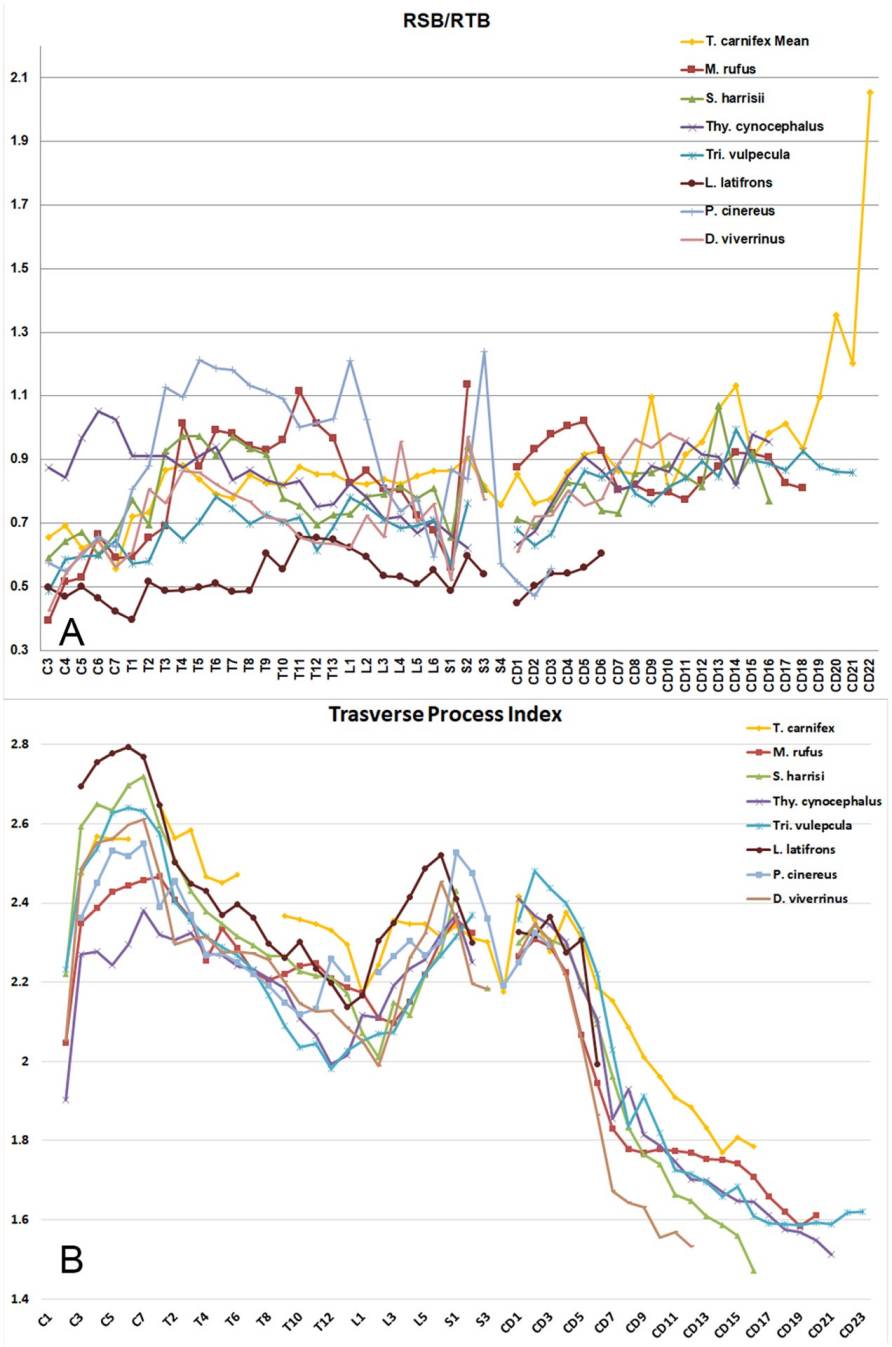
RSB/RTB and Transverse Process Index (TPI) of axial skeleton. (A) RSB divided by RTB; and (B) the TPI. All plots include the total vertebral column (except for the axis and atlas vertebrae) both for *T*. *carnifex* the other marsupials studied. Gaps in the curve relate to variation in the number of sacral vertebrae present.

When we compared the relative magnitude of RSB and RTB in each vertebra of each species we found that, in general, the axial columns of most taxa studied were more strongly braced against transverse stresses (i.e. value <1, Fig 7A). A notable exception to this is *P*. *cinereus*, which displayed stronger sagittal bracing in the thoracic region. A proportionately higher resistance to sagittal bending is also apparent in the cervical vertebrae of *Thy*. *cynocephalus* and the proximal caudal region of *M*. *rufus*. In *T*. *carnifex*, the middle and distal caudal regions display a high degree of sagittal bending resistance, with a notable peak in the distal caudals.

**Fig 8:**
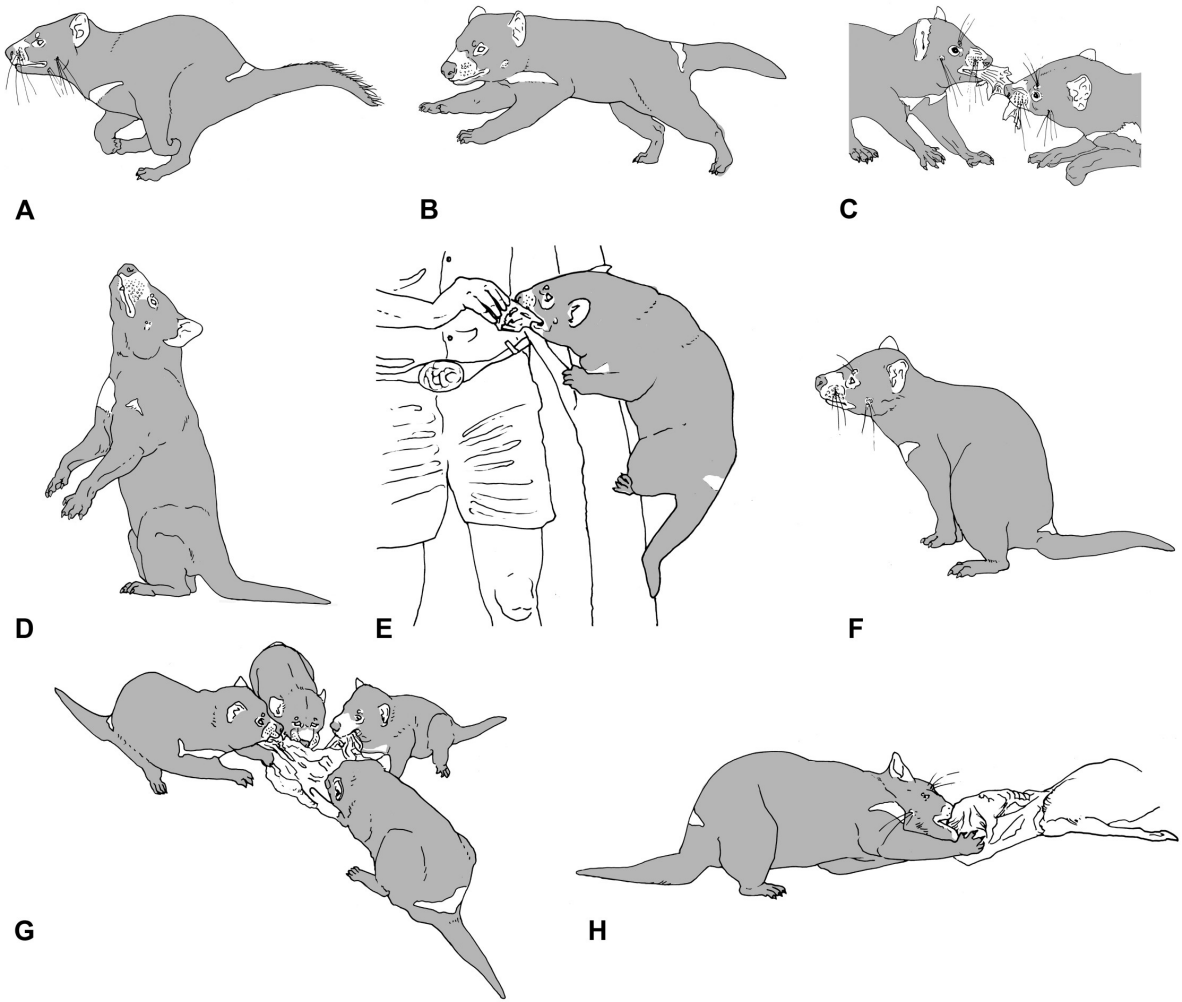
Behavioural postures of *S*. *harrisii*. Silhouettes of various behaviour and postures (modified from photos available at Wikipedia commons https://en.wikipedia.org/wiki/Tasmanian_devil) exhibited by *S*. *harrisii*. (A, B, D, F) Note the stiff lumbar region and extent of trunk and caudal flexion; (C, E, G, H) limb bracing and neck flexion when pulling back against opposing force; (H) bracing with tail and hind limbs while using forearms to hold prey while dismembering; (G) similar behaviour to (H) during group attack on a carcass; (E) using the tail as a prop while climbing (artwork by P.F. Murray).

The sacral/proximal caudal skeleton serves two major functions: anchoring musculature for both femoral (*m*. *caudofemoralis*, *m*. *gluteus superficialis*), and distal caudal (*m*. *sacrocaudalis ventralis lateralis* and *medialis*, *m*. *sacrocaudalis dorsalis medialis* and *lateralis*, *m*. *intertransversarius dorsales* and *ventrales caudae*) flexion/extension. Caudal centrum length peaked around the transitional vertebra in all species except *T*. *carnifex* and *Thy*. *cynocephalus*, in which the peak is shifted distally (Fig 3A). Caudal bending resistance was most strongly expressed in *M*. *rufus*, less so in all other species (Fig 3C and 3D). This appears to correlate with tail use in the extant species. *Macropus rufus* not only uses the tail as a counter balance in bipedal hopping and to support the body weight in pentapedal bounding but also when standing on the tail in male-male combat. *Sarcophilus harrisii* uses the tail as a brace or strut when dismembering a carcass and also when climbing (Fig 8E), *Tri*. *vulpecula* uses its prehensile tail for stability when climbing. Archival film footage of the quadrupedal *Thy*. *cynocephalus* in captivity (https://aso.gov.au/titles/historical/tasmanian-tiger-footage/clip1/) suggests only a passive flexing of the tail during locomotion. The pattern of caudal bending resistance in *T*. *carnifex* most closely resembled that of *S*. *harrisii*. In *T*. *carnifex* all the aforementioned axial features point towards a strong but rather inflexible lower back and a strong tail base of limited mobility. Further clues to posture and movement are suggested by the structure of the pelvic region.

The Transverse Process Index (TPI, displayed in Fig 7B) was measured for the entire axial skeleton of all taxa studied. In general the curves for all taxa studied group very closely. Youlatos [31] used this index to distinguish prehensility in tails of carnivorans, noting that distal caudal vertebrae were more robust in those exhibiting prehensility. The distal caudal vertebrae of *Tri*. *vulpecula* (the only taxon with a prehensile tail included in this study) are not differentiated from the other taxa studied in the TPI plot, however there is a distinct peak in the proximal caudals. Damage to the transverse processes on the preserved proximal caudal vertebrae of *T*. *carnifex* as well as missing distal caudals make it difficult to infer too much from its position on the plot. It is worth noting that the TPI for the middle caudal region plots higher than any other taxon studied. It would be difficult to conclude that this related to prehensility in *T*. *carnifex* but may indicate that the tail was being actively used in another role.

### Sacrum: Description and comparison

The sacrum of *T*. *carnifex* comprises three to four fused vertebrae in adult animals, two in juveniles and young, the third and subsequent sacral vertebrae fuse from the proximal caudals as animals mature (Fig 6I–6L).

**Fig 9:**
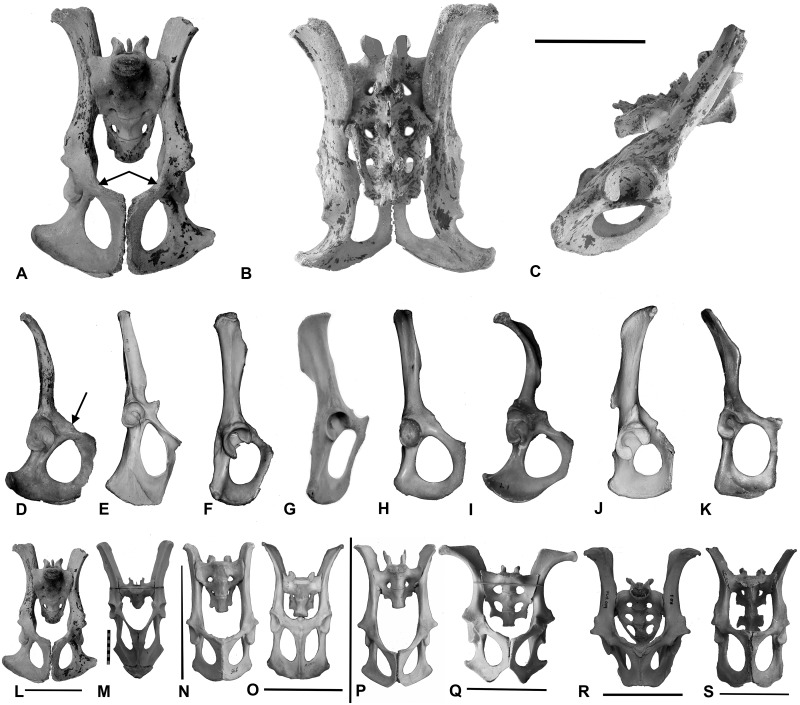
Pelves of taxa in this study. Pelvis of *Thylacoleo carnifex* (A) ventral view; (B) dorsal view; and (C) lateral views. Scale bar equals 100 mm. Right side lateral view of pelves of: (D) *T*. *carnifex*; (E) *M*. *rufus*; (F) *S*. *harrisii*; (G) *Thy*. *cynocephalus*; (H) *Tri*. *vulpecula*; (I) *L*. *latifrons*; (J) *P*. *cinereus*; and (K) *D*. *viverrinus*] showing the relative proportions of the ilium and ischium and the nature of the acetabulum, all scaled to the same overall length. Black arrows indicate attachment area for epipubics. Ventral aspect of the pelves of (L) *T*. *carnifex*; (M) *M*. *rufus*; (N) *S*. *harrisii*; (O) *Thy*. *cynocephalus*; (P) *Tri*. *vulpecula*; (Q) *L*. *latifrons*; (R) *P*. *cinereus*; and (S) *D*. *viverrinus*, scale bars equal 100 mm except in (S) where it equals 30 mm.

Each vertebra supports a well-developed dorsal spine. These spines are broken above the base in most specimens. Where preserved that of the first sacral is the tallest while that of the fourth is the shortest. These spines and the adjacent articular processes are the site of origin of the caudal extensors, *m*. *sacrocaudalis dorsalis medialis* and *lateralis*, which raise the tail. The dorsal surface of the sacrum and the adjacent medial aspect of the ilium along with the transverse processes of the proximal caudal vertebrae are the sites of origin of the abductor muscles, the *m*. *intertrans-versarii dorsales* and *ventrales caudae*, responsible for the lateral movement of the tail [28]. The sacral dimensions are listed along with those of *Thy*. *cynocephalus*, *S*. *harrisii*, *Tri*. *vulpecula and M*. *rufus* in Table 3. The degree of taper in the sacrum of *T*. *carnifex* was the least of all the species used in this comparison. The dorsal surface area expressed either relative to pelvic width or length is the largest of all species. An opposing set of muscles, *m*. *sacrocaudalis ventralis medialis* and *lateralis*, arises from the ventral surface of the sacrum and are responsible for ventrolateral tail flexion. The articulation with the ilium of the pelvis is shown in Fig 9A–9C. It is crescent shaped, deeply pocketed and buttressed caudally. It spans the first and second sacral vertebrae.

**Table 3:** Pelvic proportions, width/length, sacral taper, surface area of sacrum relative to pelvic length and width for *T*. *carnifex*, *Tri*. *vulpecula*, *S*. *harrisii*, *D*. *viverrinus P*. *cinereus*, *L*. *latifrons*, *Thy*. *cynocephalus* and *M*. *rufus*.

Species	n	Width at acetabula / pelvic length	Ischial length / pelvic length	Sacral taper anterior-posterior width/length	Sacral area/pelvic length	Sacral area/ width at acetabula
*T*. *carnifex*	3	0.45	0.32	0.30	48.58	108.74
*Tri*. *vulpecula*	2	0.56	0.35	0.54	11.90	21.14
*S*. *harrisii*	3	0.49	0.34	0.49	21.14	43.08
*D*. *viverrinus*	1	0.52	0.38	0.43	15.36	29.80
*P*. *cinereus*	2	0.58	0.31	0.39	34.11	58.72
*L*. *latifrons*	3	0.58	0.37	0.37	36.13	62.56
*Thy*. *cynocephalus*	4	0.45	0.42	0.34	12.54	39.25
*M*. *rufus*	2	0.46	0.44	0.73	36.75	79.63

The sacro-iliac angle in *Thylacoleo* is approximately 40° (Fig 2B). This is high compared with most marsupials studied, *Tri*. *vulpecula* 20°, *P*. *cinereus* 15°, *L*. *latifrons* 30°, *S*. *harrisii* 30°, *D*. *viverrinus* 25°, and is more similar to *Thy*. *cynocephalus* 40°, but not as extreme as *M*. *rufus* 60°. In *T*. *carnifex* the higher sacro-iliac angle and a slightly more posterior position of the sacro-iliac joint serves to expose more of the medial surface of the ilium, the site of attachment of the *m*. *erector spinae*. The caudal shift is similar to *P*. *cinereus* but not as pronounced as in *M*. *rufus*.

### Pelvis: Description and comparison

The pelvis, comprising the ilium, ischium and pubis is illustrated in Fig 9A–9C. The distance between the left and right acetabula measured at their dorsal border (hip width) along with pelvic length (ilium + ischium) is expressed as a pelvic slenderness ratio (width/length) in Table 3.

*Thylacoleo carnifex* is relatively slender hipped (width across acetabula / overall pelvic length), most closely equating in proportions to *S*. *harrisii*, *Thy*. *cynocephalus* and *M*. *rufus*. The ischial length to overall pelvic length is indicative of the gearing of the hamstrings (*m*. *semimembranosus*, *m*. *semitendinosus*, *m*. *adductor magnus*, *m*. *biceps femoris*), the higher the value the greater the leverage to the extensors of the hip and retractors of the femur. The value for *T*. *carnifex* is most similar to that of *P*. *cinereus*. The marked lateral flaring of the ischial tuberosity is similar to that observed in *L*. *latifrons* increasing the mechanical advantage of the adductors.

The acetabulum is formed at the junction of the ilium, ischium and pubis. It is moderately deep, buttressed anteriorly and posteriorly by a thickened rim. In contrast to the condition in *S*. *harrisii* and *Thy*. *cynocephalus*, the dorsal margin is weakly excavated and similar to that in *L*. *latifrons* and allows some abduction of the femoral head (Fig 9C).

The ilia are dorsoventrally flattened and strap-like with a moderate lateral flare to the anterior end marking the origin of the *m*. *sartorius*, a flexor of the *hip*. This lateral flaring is similar to that of *P*. *cinereus* but not as exaggerated as that seen in the wombat *L*. *latifrons* (Fig 9Q) qv. also *Vombatus ursinus* [Common Wombat], in Elftmann [38]. The flared anterior portion of the ilium also serves as the site of origin of the lateral division of the *m*. *longissimus lumborum* and *m*. *iliocostalis lumborum*, extensors and fixers of the vertebral column. A robust ovoid tuberosity, the iliopectineal eminence and origin of the *m*. *rectus femoris*, a flexor of the hip and extensor of the knee, lies adjacent to the acetabulum and bordering a groove for the *m*. *ilio-psoas*, a deep flexorof the hip but also a flexor of the back when the leg is stationary.

The ischium provides the lever arm for the hamstrings and the hindlimb adductors. In *T*. *carnifex* the dorsal ramus lies parallel to the longitudinal axis of the sacrum, and in comparison to the ilium is relatively short and robust, its length is approximately equal to that portion of the ilium lying between the acetabulum and the caudal end of the sacro-iliac joint. The ratio of ischium length to pelvic length (ischium + ilium) is 0.32 (n = 5), similar to *P*. *cinereus* 0.31 (n = 2). The vertical ramus is broad, descending in an arc to the short pubic symphysis. The pubic symphysis length /ischial length (1:1) in *T*. *carnifex* is similar to that of all other species with the exception of *L*. *latifrons* (0.6:1).

The medial borders of the ischium and pubis define the obturator foramen which in *T*. *carnifex* is pear-shaped with the long axis parallel to the vertebral column, similar to that of *Tri*. *vulpecula* (Fig 9H and 9P). The dorsal border of the ischium ends caudally and dorsally in a marked tuberosity marking the origin of the *m*. *semitendinosus*, a flexor of the knee. Its lever arm (distance from centre of acetabulum/femur length 0.27 n = 2) is similar to *P*. *cinereus* (0.25 n = 2).

A tuberosity on the antero-ventral border of the pubis (Fig 9A and 9D) marks the site of attachment of the epipubic bones. Epipubic dimensions and proportions are shown in Table 4. The epipubics of *T*. *carnifex* are proportionally similar to *L*. *latifrons* and *S*. *harrisii*.

**Table 4:** Epipubic bone dimensions and proportions for *T*. *carnifex*, *Tri*. *vulpecula*, *S*. *harrisii*, *Thy*. *cynocephalus*, *D*. *viverrinus P*. *cinereus*, *L*. *latifrons*, and *M*. *rufus*.

Epipubics Species	Length mm	Proximal width mm	Distal width mm	Prox. width/length x 100	Distal width/length x100
*T*. *carnifex*	60.44	27.53	7.79	45.55	12.88
*Tri*. *vulpecula*	23.98	13.26	2.98	55.29	5.04
*S*. *harrisii*	48.25	15.95	5.92	33.05	12.27
*Thy*. *cynocephalus*	52.29	14.70	4.26	52.29	8.15
*D*. *viverrinus*	31.13	9.69	1.73	31.12	5.55
*P*. *cinereus*	69.67	26.13	13.06	37.50	18.74
*L*. *latifrons*	59.49	26.61	8.32	44.73	13.98
*M*. *rufus*	90.45	10.52	6.54	11.63	7.23

### Clavicle: Description and comparison

**Fig 10:**
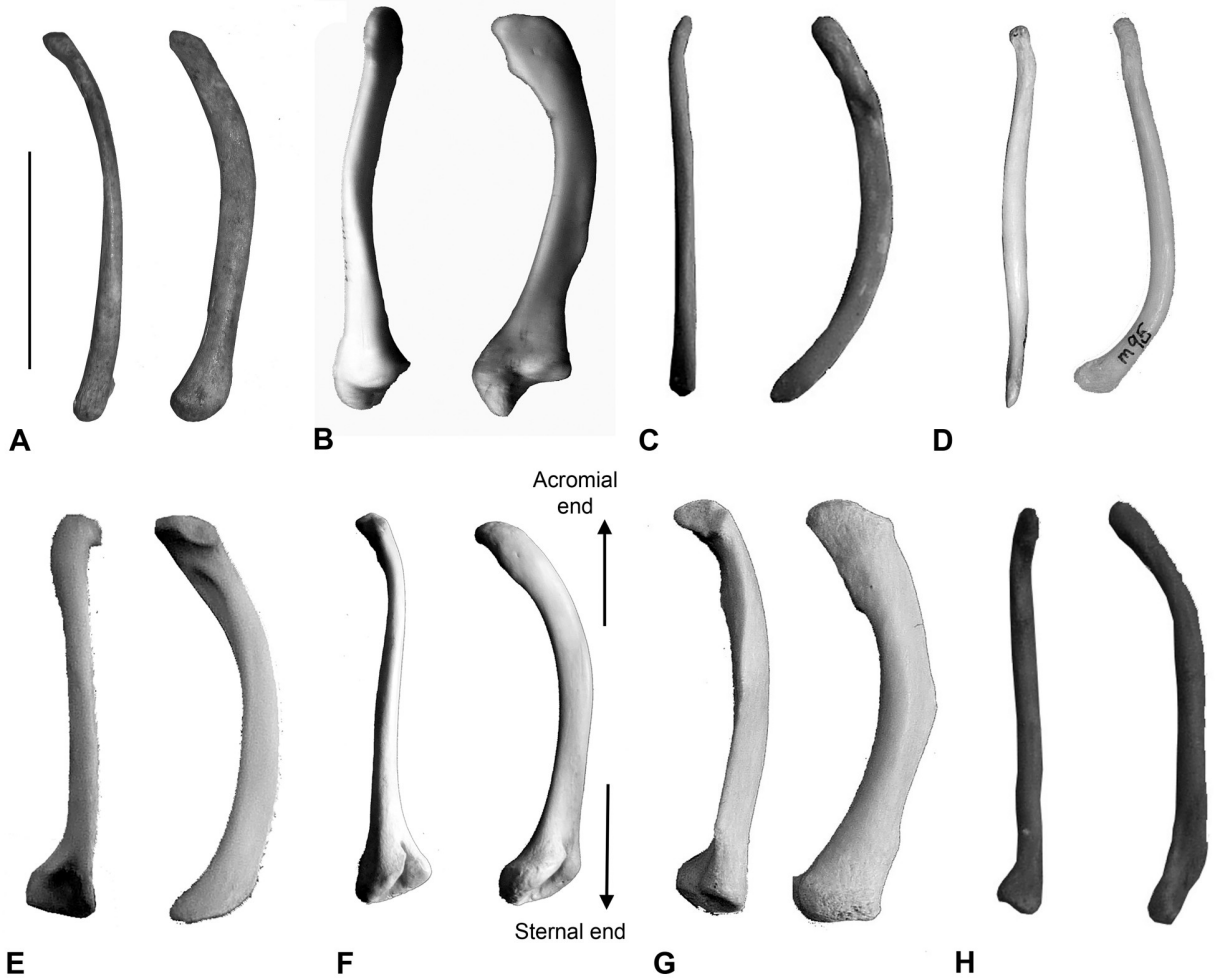
Clavicles of taxa studied. Clavicles of (A) *T*. *carnifex*; (B) *M*. *rufus*; (C) *S*. *harrisii*; (D) *Thy*. *cynocephalus*; (E) *Tri*. *vulpecula*; (F) *L*. *latifrons*; (G) *P*. *cinereus*; and (H) *D*. *viverrinus*, all scaled to be the same size as *T*. *carnifex*.

The clavicle of *T*. *carnifex* describes a simple asymmetric external curvature similar to that of *S*. *harrisii* (Fig 10C). The distal or acromial end is slender with only weakly developed trapezoidal and deltoid muscle scars. Clavicle resistance to both dorsal and lateral loading is listed in rank order for a range of species in Table 5. Clearly when calculating bending strength against clavicle length *T*. *carnifex* ranks with the arboreal *P*. *cinereus*, however the clavicle of *P*. *cinereus* (Fig 10G) has a broad flattened acromial end, a well-defined ridge flaring in mid-shaft marking the insertion of the pectoralis musculature, features indicative of a species which uses the forelimb in climbing [39]. In contrast the form of the clavicle in *T*. *carnifex* is consistent with the bracing of a shoulder girdle in which scapula rotation is predominantly in the sagittal plane.

**Table 5:** Clavicle resistance to dorsal (bh^2)^ and lateral (hb^2^) bending for *M*. *rufus*, *T*. *carnifex*, *P*. *cinereus*, *L*. *latifrons*, *Thy*. *cynocephalus*, *Tri*. *vulpecula*, *S*. *harrisii*, *D*. *viverrinus*.

Taxon	Length (l) mm	Min. dia. mid shaft (b) mm	Max. dia. mid shaft (h) mm	bh^2^/ 100	bh^2^/l	hb^2^/100	hb^2^/l
*M*. *rufus ♂*	102.45	6.45	11.13	7.99	7.80	4.63	4.52
*T*. *carnifex*	99.64	5.04	10.87	5.96	5.98	2.76	2.77
*P*. *cinereus ♂*	54.30	4.46	8.08	2.91	5.36	1.61	2.96
*L*. *latifrons*	73.38	3.87	6.63	1.70	2.32	0.99	1.35
*Thy*. *cynocephalus♂*	42.18	2.32	2.83	0.19	0.44	0.15	0.36
*Tri*. *Vulpecula*	29.07	1.68	2.87	0.14	0.48	0.08	0.28
*S*. *harrisii ♂*	43.35	1.58	3.00	0.14	0.33	0.07	0.17
*D*. *viverrinus*	25.04	1.00	1.80	0.03	0.13	0.02	0.07

**Fig 11:**
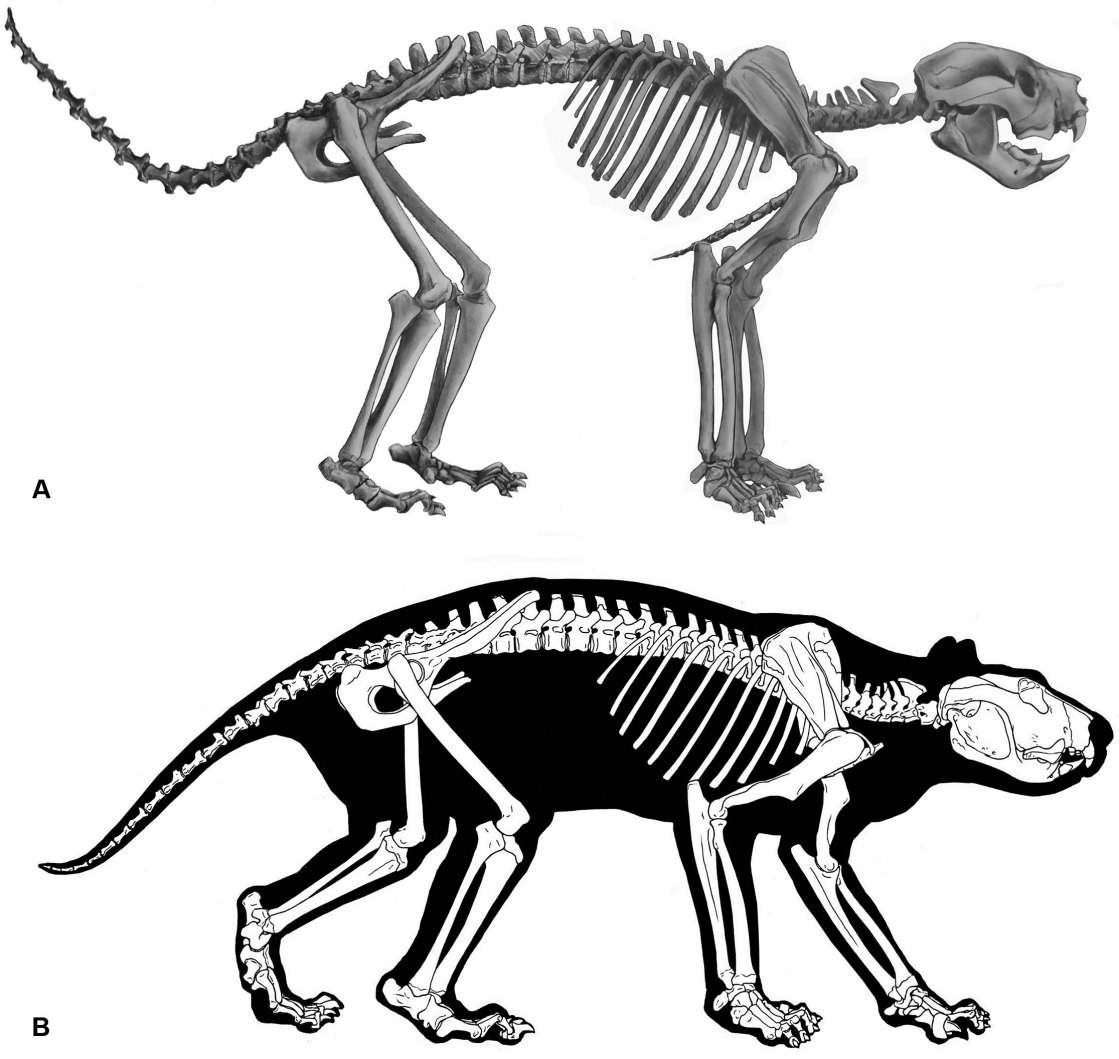
*Thylacoleo carnifex* reconstructions. (A) Reconstruction of the skeleton of *T*. *carnifex*. (B) Body outline based on examination of musculature evident in x-ray imaging of marsupials Vogelnest and Allen [11].

We have combined all the aforementioned features along with previously published accounts to re-assemble the skeleton of *T*. *carnifex* (Fig 11).

## Discussion

Placing the skeletal elements of *Thylacoleo carnifex* in correct anatomical sequence posed little challenge; reconstructing posture and biomechanical constraints to behaviour proved more challenging. Behavioural inferences are based on skeletal comparisons with extant marsupials in which behaviour is known. In this study the dasyurid *Sarcophilus harrisii* emerged as the closest functional analogue. Of particular interest were those portions of the skeleton not previously recognised or described, the caudal skeleton and clavicles, their structure and functional implications.

### Functional interpretation of the caudal skeleton (tail)

The general form of the caudal skeleton in *T*. *carnifex* exhibited similarity to that of the dasyuromorphs *S*. *harrisii* and *Thylacinus cynocephalus*. These species carry the proximal portion of the tail in a horizontal position when moving quadrupedally. *Sarcophilus harrisii* uses the tail and hind limbs in tripod fashion as a brace when tearing apart carrion or as a prop when climbing (Fig 8). We suggest that *T*. *carnifex* was capable of using the tail in a similar fashion. In support of this scenario we interpret the relatively large sacral area, broad lateral processes of the proximal caudal vertebrae in *T*. *carnifex* as indicative of a strong muscular tail base. These processes are sites of attachments for muscles controlling both tail flexion and abduction. Because the pre- and post- zygapophyses of the proximal caudal vertebrae Cd1–7 would have greatly constrained the range of lateral and dorsal flexion and extension, any flexibility of the tail would largely have been confined to the region beyond the transitional vertebra from Cd10 to the tip of the tail.

The presence of chevron bones across the ventral caudal junctions is a feature common to all Australian marsupials we examined qv. also X-rays, Vogelnest & Allen [11]. The nature of these chevrons provides a further clue to tail use. In marsupials the lateral aspect of the chevrons are sites of insertion of tendons of both the *m*. *flexor caudae lateralis* and the *m*. *flexor caudae medialis* [23]. Contraction of these muscles on alternate sides contributes to lateral flexion of the tail. Chevrons in species with highly mobile tails (e.g. phalangerids, macropodids and some small dasyurids) exhibit well developed chevron keels providing an enhanced area for tendon attachment [11]. Keels appear less well developed in species with shorter robust tails such as *S*. *harrisii*. The weak development of these keels in *T*. *carnifex* would seem to indicate a similar limited capacity for strong lateral flexion. Equal loading of the lateral muscle tendon system would, however, serve to hold the base of the tail in compression such that it would appear relatively stiff and inflexible when moving but would still allow dorsoventral flexion in the distal caudal vertebrae. The eversion of the hind feet and the mobile fibula in *T*. *carnifex* [9] are typical of species adapted to moving or standing on uneven surfaces [40], features also evident in *S*. *harrisii* clearly illustrated in its behaviour when dismembering a carcass or climbing (Fig 8). Here the combined use of tail and hind limbs provides the stability necessary to free the forelimbs for manipulation of prey or raising the body when climbing. We suggest that *T*. *carnifex* was capable of using the hind limbs and tail in similar fashion.

### Functional implications of the axial skeleton

Inflexion of the neck and curvature of the thoracic spine in *T*. *carnifex* is apparent in both juveniles and adults. This similarity in spinal curvature to that of dasyurids qv [11] would suggest a convergence in function. Retention of an ancestral condition is unlikely given that diprotodontians and dasyuromorphians diverged early in the evolution of australidelphian marsupials [41].

The vertebral column is a dynamic structure, so any straightening of a sagittal curvature would contribute to stride length, while any rotational flexing would relate to the torque produced by alternate use of right and left limbs in a transverse-gait. In *T*. *carnifex* the relatively straight and rigid lumbar region with its torque-resisting, saddle-shaped zygapophyseal articulations, broad but relatively short, non-overlapping lateral processes and neural spines with their caudal inclination suggest a relatively inflexible lower back. These are characters shared with *P*. *cinereus* and *S*. *harrisii*. The latter two species utilise a rather awkward transverse gait at slow speed with any rotational torque occurring around the lumbar–thoracic junction. They utilise a half bound at higher speeds with no obvious evidence of sagittal flexing or rotational torque, their vertebrae, held in compression, making limited if any contribution to stride length. This stands in contrast to more agile arboreal/scansorial forms such as *Tri*. *vulpecula*, *D*. *viverrinus*, the cursorial *Thy*. *cynocephalus* or the fossorial *L*. *latifrons*, all species exhibiting anticlinicity (cranial inclination of the lumbar spinal process) capable of exerting greater control over a more flexible lower back.

The resistance curve for *T*. *carnifex* falls into Slijper’s category Type 1b [17]: animals that are at times quadrupedal and at times stand or sit on their hind legs, such as the American black bear, *Ursus americanus*. The more caudally positioned sacro-iliac joint and high sacro-iliac angle in *T*. *carnifex* providing greater ilial leverage to erector spinae and femoral muscles are consistent with an animal capable of standing or sitting on its hind feet.

The pattern of resistance to both lateral and sagittal centrum bending is similar in the trunk vertebrae of all species studied. Species differences are largely confined to the cervical and caudal vertebrae. *Thylacoleo carnifex*, *M*. *rufus*, *Thy*. *cynocephalus* and *S*. *harrisii* all exhibit resistance peaks around the cervical–thoracic junction. With the exception of *T*. *carnifex*, all tailed species also exhibit resistance peaks around the transitional caudal vertebrae. Excluding *M*. *rufus*, all extant taxa studied also demonstrate a sharp dip in resistance to sagittal bending at the sacro-caudal interface. In contrast, in *T*. *carnifex* caudal RSB and RTB peaks around the proximal caudals. Among all the species used in this study the overall stress curves for *T*. *carnifex*, *S*. *harrisii*, *M*. *rufus* and *Thy*. *cynocephalus* show the greatest similarity in pattern with resistance peaks around the shoulder and hip region of the column, a pattern that is typical of terrestrial quadrupeds in which a higher level of sagittal stress is exerted by the hind limbs and, to a lesser extent, the forelimbs. In contrast, the major RSB in the vertebral columns of the arboreal *P*. *cinereus* and scansorial *Tri*. *vulpecula* and *D*. *viverrinus* is confined to the lumbar-sacral area, indicative of the loading by the hind limbs when climbing, but no peak around the forelimbs.

The resistance to sagittal flexion in the tail base of *T*. *carnifex*, although greater than that of the arboreal and scansorial species, falls well short of that exhibited by the large red kangaroo, notwithstanding similarity in body weight. This is not surprising given that large macropodids use their tails as a fifth leg during slow locomotion [24, 42]. Still, the fact that the resistance is significantly higher than in the non-saltatorial species studied suggests that *T*. *carnifex* was actively employing its tail in a way that differs to these other marsupials. Peaks in the relative magnitude of RSB (when compared to RTB) suggest that the tip of the tail of *T*. *carnifex* was used for a different role than that employed by all other species studied (see Fig 7A). The peaked resistance may indicate that *T*. *carnifex* was holding its tail up in the air, possibly for use as a prop when climbing or perhaps even in signalling, although we found no analogues for this amongst extant marsupials [43]. The TPI curve for *T*. *carnifex* (Fig 7B), although incomplete, also suggests that its tail was adapted to a different role than those of the other species studied. Youlatos [31] used this index to differentiate taxa with prehensile tails and in our plot *T*. *carnifex* groups closest to *Tri*. *vulpecula*, the only taxon studied with a prehensile tail. The data for *T*. *carnifex* is incomplete due to missing or damaged processes on the specimens available for study. Whilst we think it unlikely that the tail was prehensile, the TPI may bolster support that *T*. *carnifex* used its tail in novel behaviours not seen in other Australian marsupials.

The sacral/proximal caudal skeleton serves two major functions: anchoring musculature for both femoral and distal caudal flexion/extension. Caudal centrum length peaked around the transitional vertebra in all species. Resistance to bending was most strongly expressed in *M*. *rufus*, less so in all other species (Fig 3C and 3D). This appears to correlate with tail use in the extant species. *Macropus rufus* not only uses the tail as a counter balance in bipedal hopping and to support the body weight in pentapedal bounding but also when propping on the tail in male-male combat [44]. *Sarcophilus harrisii* uses the tail as a brace or strut when dismembering a carcass and also when climbing [45, 46]. *Trichosurus vulpecula* uses its prehensile tail for stability when climbing [47]. Archival film footage of *Thy*. *cynocephalus* in captivity suggests only a passive flexing of the tail during locomotion [48]. The pattern in *T*. *carnifex* most closely resembles that of *S*. *harrisii*. In *T*. *carnifex* all the aforementioned axial features point towards a strong but rather inflexible lower back, a strong tail base of limited mobility. Further clues to posture and movement are suggested by the structure of the pelvic region.

### Functional implications of the appendicular skeleton

#### The pectoral girdle

Koalas have relatively long (forelimb/hindlimb 96% *cf T*. *carnifex* 94%), high-geared forelimbs (radial/humeral ratio 114% *cf T*. *carnifex* 115% [4]) and are schizodactylous with sharp, recurved unguals. These features relate to the manner in which they climb, hauling themselves up trees alternately using the fore- and hind-limbs, moving the hind limbs in a bounding motion. The schizodactylous grip in *P*. *cinereus* has been viewed as functionally similar to the development of the first digit and pisiform in the manus of *T*. *carnifex* [7]. Finch [4] also noted the biomechanical similarity to koalas in the manus and forelimb proportions of *T*. *carnifex*, while Wells et al. [8] even used its inferred climbing ability to propose a leopard-like niche. This mode of climbing employed by *P*. *cinereus* requires a well-braced pectoral girdle and, to this end, koalas have evolved robust clavicles.

Clavicle morphology is directly related to locomotor behaviour serving to brace the pectoral girdle against forelimb loading [49]. Clavicles are particularly well developed in arboreal and fossorial species. All Australian marsupials with the exception of the Peramelidae (bandicoots) have clavicles. The clavicles of *T*. *carnifex*, although robust and hence compression resistant, exhibit only a simple curvature. This is a feature associated with terrestrial quadrupeds in which scapular rotation is in the sagittal plane and is consistent with the rectangular form of the scapula more typical of cursors [4, 5].

The clavicle of *T*. *carnifex* is most similar in form to that of *S*. *harrisii* but in inferred strength (length/cross-sectional area) to that of *P*. *cinereus*. The clavicle shows a proportionally similar resistance to bending stresses to that seen in *P*. *cinereus* (see Table 5). However, the clavicles of *T*. *carnifex* lack the strong tubercular development observed in *P*. *cinereus*, *L*. *latifrons* and *M*. *rufus*, suggesting they serve as simple compression struts stabilising the shoulder. Although these features suggest a strong bracing of the pectoral girdle, they do not of themselves reflect on climbing ability.

#### The pelvic girdle

In lateral aspect the pelvis of *T*. *carnifex* somewhat resembles that of *L*. *latifrons*. The acetabulum of *T*. *carnifex* is similar in form to both *L*. *latifrons* and *P*. *cinereus* with slight excavation and weak lipping of the dorsal margin allowing some abduction of the femur. Although the ability to abduct the femur occurs in most marsupials, it is accentuated in short-legged, fossorial forms like wombats when burrowing and in arboreal forms when grasping narrow branches, femoral abduction is also important in saltatorial terrestrial species when carrying large pouch young. It is compensated by medial rotation of the crus, realigning the tarsi in the sagittal plane [50] and development of the *m*. *adductor magnus*. Small arboreal forms also abduct the femur. In the absence of evidence for fossoriality or saltatorial locomotion, we infer that the pelvic morphology of *T*. *carnifex* is most likely related to its climbing ability [7, 8, 9, 51]. The distally flared ischium of *T*. *carnifex* and associated adductor development may suggest either adaptation to a crouching posture, or allometric scaling associated with a trunk-hugging form of climbing, as seen in *P*. *cinereus*.

### Functional implications of limb morphology

Certainly Wells and Nichol [7] and Wells et al. [9] viewed the hind-foot structure in *T*. *carnifex* as an adaptation to movement over uneven surfaces and evidence of climbing ability. However, the wide geographic distribution of fossil material and body weight estimates of up to 130 kgs [15] raise questions about the broad availability of suitable tree habitat to support an arboreal lifestyle, notwithstanding that no one had ever proposed that *T*. *carnifex* was an habitual tree dweller. Evidence of climbing ability emerged from an unexpected source, the treeless Nullarbor Plain. Arman and Prideaux [51] studied the large collection of bones accumulated in Tight Entrance Cave, a deep pitfall site and den that included the remains of a number of *T*. *carnifex* specimens. They noticed a large number of scratch marks on the steeply sloping surfaces within the cave. In a detailed actualistic study comparing digit spacing and unguis shape among extant marsupials they, by a process of elimination, were able to attribute the deeper scratch marks to those made by the manus of *T*. *carnifex* when climbing in and out. They suggest that *T*. *carnifex* were “excellent climbers and reared young in caves”. Finch and Freedman [6] noted the similarity in the radial-humeral ratio to that of *Tri*. *vulpecula* (R/H 112%) but concluded that the similarity was “not likely to indicate a common climbing habit but to imply cursorial locomotion and the use of the forelimb in handling food”. Figueirido et al. [52] in their comprehensive comparative study of elbow joint morphologies concluded that *T*. *carnifex* exhibited forearm mobility intermediate between that of wombats and arboreal species, all of which require a high degree of elbow mobility.

Herein lies a paradox, we have a mammal with a high-geared (prox/distal ratio *sensu* Hildebrand & Goslow [18]) yet powerful mobile forelimb and a low-geared mobile hindlimb. The evidence that Finch and Freedman [6] advanced in support of cursoriality stemmed largely from the nature of the scapula with its almost equal supra- and infra-spinous fossae, a feature shared with cursors. However, as Davis [53] notes, when discussing the glenohumeral articulation, contrary to statements made in the literature, “in carnivores many of the short powerful scapular muscles are far more important in fixing the joint than in producing movement”. This may well be the case in *T*. *carnifex*. Wells and Nichol [7] concluded that the forelimb structure, particularly that of the manus, provided an efficient and powerful mechanism for grasping, holding and manipulating prey.

Finch and Freedman [6] noted the similarity in limb indices (forelimb/hindlimb *S*. *harrisii*, 96%; *T*. *carnifex* 94%) and scapula proportions to those of *S*. *harrisii* and concluded that *T*. *carnifex* was a ‘slow-medium cursor, possibly capable of leaping’. Certainly the lever arm of the ilium suggests powerful hind limb flexors while the strength in the proximal portion of the tail is consistent with the stresses imposed by the extensor and abductor musculature when leaping. The rigid lumbar spine and strong lever arm for the *m*. *erector spinae* musculature, combined with a strong tail and low-geared hindlimbs, could in addition, serve to anchor or brace the body while freeing the forelimbs for use when climbing and/or manipulating prey. Wells et al. [9] in their description of the pes noted the rotatory capability of the subtalar, tibia/ fibula joint which, when combined with the relatively narrow pes with its scimitar-like unguals, they suggested could serve to anchor the hind feet when manipulating prey.

It has been argued that the forelimb and manus play a major role in prey capture in *T*. *carnifex* [4, 7, 8] rather than the canines which are vestigial, a reflection of its diprotodontian ancestry. Finch [4] remarked on the length of the neck relative to the thorax in *T*. *carnifex*, a feature typical of the carnivore cursors such as *Dasyurus* and *Sarcophilus*. A strong and mobile neck is essential to carnivores that typically use their teeth in prey capture. In *T*. *carnifex* the procumbent and convergent lower first incisors are sharp penetrating teeth while the upper first incisors have a smooth rounded occlusal surface. The lowers may simply have served to sever the spinal column in a penetrating neck bite and/or equally well serve to penetrate a joint capsule in the process of disabling prey or dismembering a carcass [8]. The limited gape in *T*. *carnifex* poses no challenge to this hypothesis; what is clear is that some neck/head mobility about the atlas and axis is essential to engage the very large sectorial premolars. The remainder of the cervical column is notably broad, robust and relatively restricted to flexion in the sagittal plane.

### Inferred behaviour

*Thylacoleo carnifex* has intrigued palaeontologists for more than 150 years with many hypotheses advanced to account for its niche and behaviour (qv. Table 6). In our behavioural reconstruction we begin with the generally accepted premise that *T*. *carnifex* is indeed a carnivore. This inference is based on the published accounts of the remarkable cheek dentition and associated jaw mechanics. The vestigial canines and short broad palatecombined with a limited gape and parrot-beak-like incisor configuration reflect its diprotodontian ancestry and clearly indicate that *T*. *carnifex* employed its incisor array in a way unparalleled amongst extant carnivores. A thrust with the honed puncturing tip of the paired lower incisors, against the resistance of the robust, buttressed mandibular fossa may have been employed to sever the spinal cord at the neck, sever tendons or cause massive trauma to a struggling prey during capture. The powerful forelimbs equipped with grasping hands and slashing first digit would amply serve to both to restrain a victim or a carcass. We envisage *T*. *carnifex* dismembering a carcass by piercing with the lower incisors against the resistance of the uppers using its strong neck to withstand the torsional and tensional loads involved in capturing, butchering and/or carrying large prey. We view such feeding behaviour as analogous to that exhibited by, the marsupial scavenger/predator *S*. *harrisii* whose axial skeletal structure most closely resembles that of *T*. *carnifex* amongst Australian marsupial carnivores. A reconstructed skeleton illustrating two possible standing postures within the biomechanical constraints imposed by this marsupial skeletal architecture are illustrated in Fig 11.

**Table 6:** Palaeobiology summary. A summary of the majority of palaeobiological inferences that have been made in the literature with regard to *T*. *carnifex*.

Element	Morphological feature	Inference	Reference
**Skull**	condyloid process of jaw	Carnivore	[54]
**Dentition**	incisors procumbent and together	herbivore	[55]
	no transverse incisor row	herbivore	[3]
	incisor/premolar combination	ooivore	[56, 57]
	incisor/premolar combination	omnivore	[2, 58, 59, 60, 61, 62, 63]
	slicing premolar	bone-chewer	[64, 65, 66, 67, 68, 69]
	slicing premolar	carnivore	[1, 64, 65, 70, 71, 72]
	slicing premolar	coarse browse herbivore	[73]
	slicing premolar	native melon specialist	[3]
	tooth morphology, microwear and jaw mechanics	meat specialist carnivore	[8]
	tooth morphology and powerful forearms	large prey specialist	[68, 74]
	vertical shear direction	hypercarnivory	[16]
**Cervicals**	well-developed neural crests	strong and flexible neck	[5]
**Lumbars**	U-shaped zygapophyses	torsion resistance possibly associated with bipedal activities	This paper
	similar in shape and robusticity to *Sarcophilus*	similar ‘rocking’ form of quadrupedal locomotion employed	This paper
**Axial skeleton**	RSB in cervicals and thoracics	omnivore	[5]
	morphology of neural spines	carnivore with phalangerid ancestry	[5]
	relative proportions of segments most similar to large dasyurids	some climbing ability? Similar mode of terrestrial locomotion?	This paper
	RSB throughout axial skeleton	similar to quadrupedal mammals that sometimes employ a bipedal stance	This paper
**Sacrum**	long and robust	powerful hindlimbs	[5]
	large area for attachment of caudal extensors and abductors	strong lateral movement and dorsal flexion of tail	This paper
	higher sacro-iliac angle	allows m. erector spinae to more effectively raise the front of the body off the ground	This paper
**Caudals**	most resistant to sagittal flexion in proximal caudals	used to brace against ground during prey capture/feeding	This paper
**Scapula**		not a climber, muscles well-developed for killing and feeding	[6]
**Clavicle**	bending strength relative to length most similar to *Phascolarctos*	arms being employed for more than just locomotion- climbing and/or prey capture	This paper
**Humerus**	large tuberosity for attachment of the m. triceps brachii	groups with arboreal marsupials	[75]
	Morphology of humeral trochlea	Highly mobile elbow indicating some climbing ability and use of forelimb for grasping and manipulating prey	[52]
**Ulna**	large olecranon	fast-striking forelimb	[6]
**Manus**	Unciform, cuneiform, MC V and phalangeal morphology	groups with arboreal marsupials	[75]
	claws	striking and tearing prey	[6]
	Grasping thumb	climbing	[7]
	Grasping thumb	prey capture	[8]
	Grasping thumb	prey capture	[76]
	Grasping thumb	Comparable to schizodactyly in *P*. *cinereus* for climbing	This paper
	Large claw on 1st digit	apprehension of prey	[6]
	broad, flattened MC V	fast-striking forelimb	[6]
	digits when flexed	ideal for grasping cylindrical objects such as branches	[9]
**Pelvis**	ilial morphology and post-acetabular length	most similar to Sarcophilus	[6]
	ischium angle	most similar to cursors (crouching whilst hunting)	[6]
	open acetabulum	allows great flexibility in hip joint (similar to *Phascolarctos*)	This paper
**Pes**	limited flexion and extension and capacity for inversion/eversion of ankle	similar to fossorial and scansorial species	[9]
	metatarsal, entocuneiform and tibio-talar joint morphology	limited grasping ability possibly related to climbing	[9]
	tarsus and metatarsus	most similar to vombatids	[9]
	ungual phalanges highly curved	correlates with significant climbing ability	[9]
	digits when flexed	ideal for grasping cylindrical objects such as branches	[9]
	morphology of distal tibia, tarsus and digits	groups with arboreal marsupials	[75]
	digits similar to *Trichosurus*	possibly clasping during climbing	[7]
**Other parameters**	bone strontium and zinc levels	carnivore	[77]
	low gearing of m. gastrocnemius	useful in climbing	[9]
	pedal and hindlimb morphology	may have been able to tripod with hindlimbs and tail during prey capture	[9]
	limb adaptations	grasping and climbing (leopard-like niche)	[8]
	forelimb:hindlimb ratio	cursorial or running	[6, 54]
	intralimb indices	slow-medium cursor but also similar to koala	[6]
	intralimb indices	stalking hunter	[4]
	radius:humerus ratio	slow-medium cursor and food manipulation	[6]
	powerful hindlimbs	pounced on prey	[6]
	bite marks on bones	hyaena-like scavenger	[68]
	bite marks on bones	bone chewer	[67]
	bite marks on bones	meat specialist carnivore	[76]
	scratch marks in caves	climbing ability	[51]
	body mass, brachycephaly, short back, lumbar neural spine orientation, claw of first digit	large prey specialist	[15]
	body mass	not a climber	[15]
	short metapodials and tibiae relative to prox. limb elements, short, thick trunk, large distance between bicipital tuberosity and prox end radius	bear (and *Smilodon*)-like niche, but hypercarnivorous, decreased cursorial performance	[16]
	similarity to ursids and *Smilodon*	takes large prey by short distance ambush, grappling with forelimbs whilst balancing on hindlimbs	[16]

We find much evidence in the skeletal anatomy to suggest it was capable of climbing; however, those features it shares with arboreal species are also features it shares with scansorial carnivores. The structure of its backbone, pectoral and pelvic girdles along with limb mechanics argue against a pursuit predator and point more towards a stealth or ambush predator and scavenger; a niche today occupied by *S*. *harrisii*. We suggest *T*. *carnifex*, as the apex marsupial carnivore, occupied a similar niche but scavenged/ hunted larger prey. Their ability to anchor the hind quarters using the tail as a brace freed the forelimb and manus to hold, lacerate or eviscerate struggling prey and/or to hold a carcass while severing limb elements, flesh and hide with a rearwards pull of the shoulders, neck and thorax. As the most flexible articulations in the vertebral column are found in the thorax it is possible that the arthritic damage observed in WAM 02.7.2 was likely induced by this form of behaviour either when subduing prey or when opposing the pull of other individuals at a carcass. We cannot say whether they were cooperative hunters or simply opportunists however the not uncommon occurrence of multiple adults and young in cave deposits [51] suggests a high degree of sociality and is worthy of further investigation. The dorsally flexed tail and increased RSB may indicate that the most distal third of the tail played a different role to that observed in any extant Australian marsupial.

## Supporting information

S1 Table Raw data related to all species studied in this manuscript. (XLSX)
